# SP-LCC — a dataset on the structure and properties of lignin-carbohydrate complexes from hardwood

**DOI:** 10.1038/s41597-025-05327-8

**Published:** 2025-06-13

**Authors:** Marie Alopaeus, Matthias Stosiek, Daryna Diment, Joakim Löfgren, MiJung Cho, Jarl Hemming, Teija Tirri, Andrey Pranovich, Patrik C. Eklund, Davide Rigo, Mikhail Balakshin, Chunlin Xu, Patrick Rinke

**Affiliations:** 1 Laboratory of Natural Materials Technology, Åbo Akademi University, Henrikinkatu 2, 20500 Turku, Finland; 2 Department of Physics, Technical University Munich, James-Franck-Str. 1, 85748 Garching, Germany; 3 Atomistic Modelling Center, Munich Data Science Institute, Technical University Munich, Walther-von-Dyck-Straße 10, 85748 Garching, Germany; 4 Department of Applied Physics, School of Science, Otakaari 1, Aalto University, 02150 Espoo, Finland; 5 Department of Bioproducts and Biosystems, School of Chemical Engineering, Aalto University, Vuorimiehentie 1, 02150 Espoo, Finland; 6 Laboratory of Molecular Science and Engineering, Åbo Akademi University, Henrikinkatu 2, 20500 Turku, Finland

**Keywords:** Biopolymers, Polymer characterization, Structure elucidation

## Abstract

Lignin-carbohydrate complexes (LCCs) are bioproducts with high potential as alternatives for petrochemicals. However, the complex structure and the lack of protocols for high-yield production limit their usage. Herein, we present data collected from a comprehensive artificial intelligence (AI)-guided optimization of the AquaSolv Omni (AqSO) biorefinery process targeting high-yield production of LCCs. The resulting database, termed SP-LCC, includes structural information extracted from nuclear magnetic resonance measurements (NMR) and data on the molar mass distribution, antioxidant activity, glass transition temperature, thermal degradation, and surface tension. In total, we collected data for 95 LCC-containing samples isolated for different AqSO process conditions. SP-LCC provides a holistic dataset for LCC development, materials understanding, and exploiting the LCC valorization potential. Furthermore, SP-LCC provides valuable data for training machine learning models for further optimization of biorefineries outside the scope of AqSO.

## Background & Summary

Lignin is the most abundant aromatic biopolymer and is an attractive candidate for replacing fossil-based feedstocks^1–4^. In traditional biorefineries, lignin has mainly been regarded as a low-value by-product, because its complex structure poses challenges to valorization, and has not been used to its full potential^5–9^. For example, Kraft lignin has been burnt primarily as a low-value energy source^10^. Lignin largely consists of the three monolignols *p*-coumaryl (H), conifernyl (G), and sinapyl (S) alcohol, which are randomly arranged and crosslinked within the structure^1,11,12^. This randomness is one of the main factors contributing to the structural complexity of lignin, which arises chiefly due to the nonenzymatic nature of the last step of the lignin biosynthesis^1^. In addition, the structural features of lignin are heavily influenced by the raw material, type of pretreatment, isolation method, fractionation method, and different process conditions^2,4^.

**Fig. 1:**
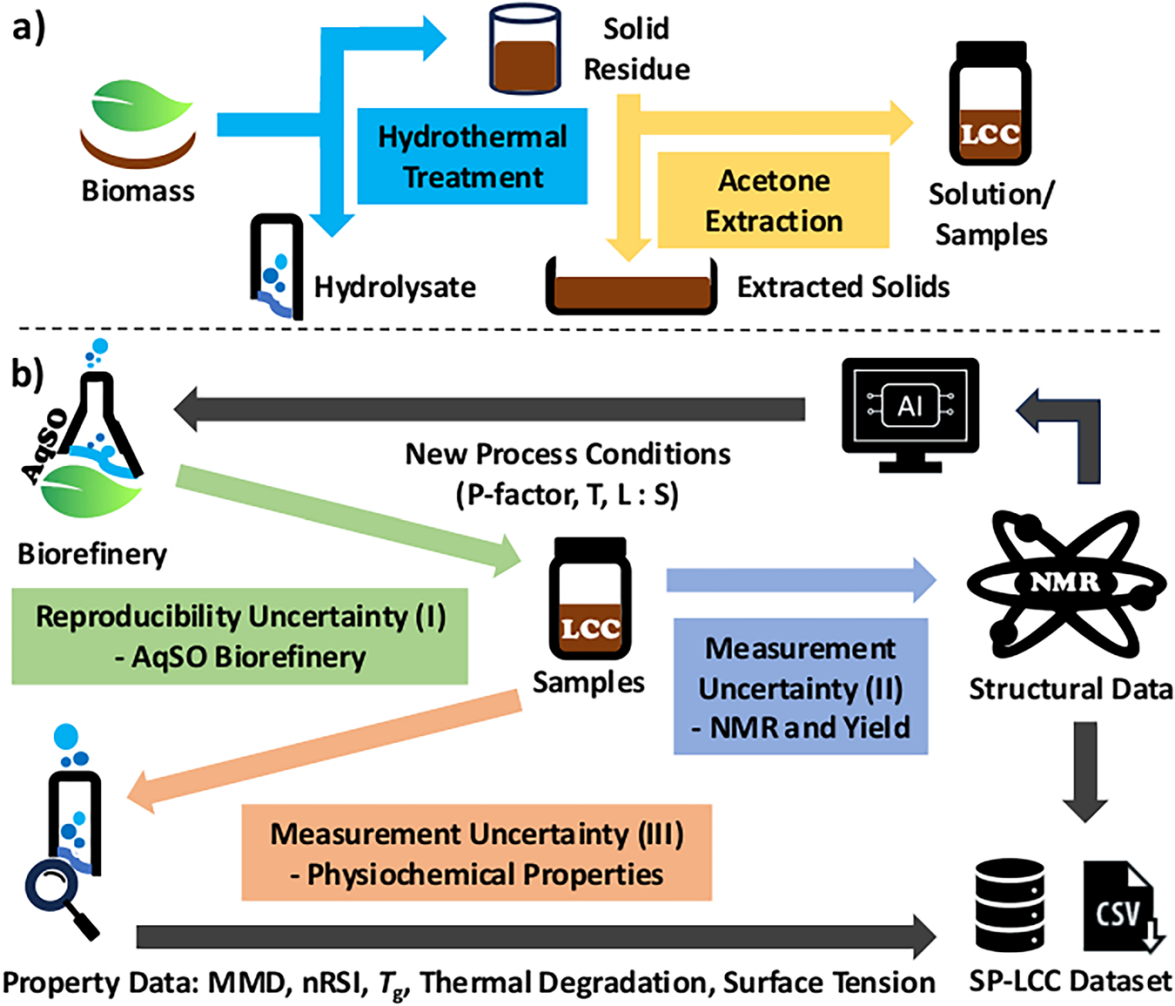
Schematic overview of the workflow for the SP-LCC dataset, where (**a**) shows the process steps of the AqSO biorefinery and (**b**) the collection of the experimental data.

**Fig. 2:**
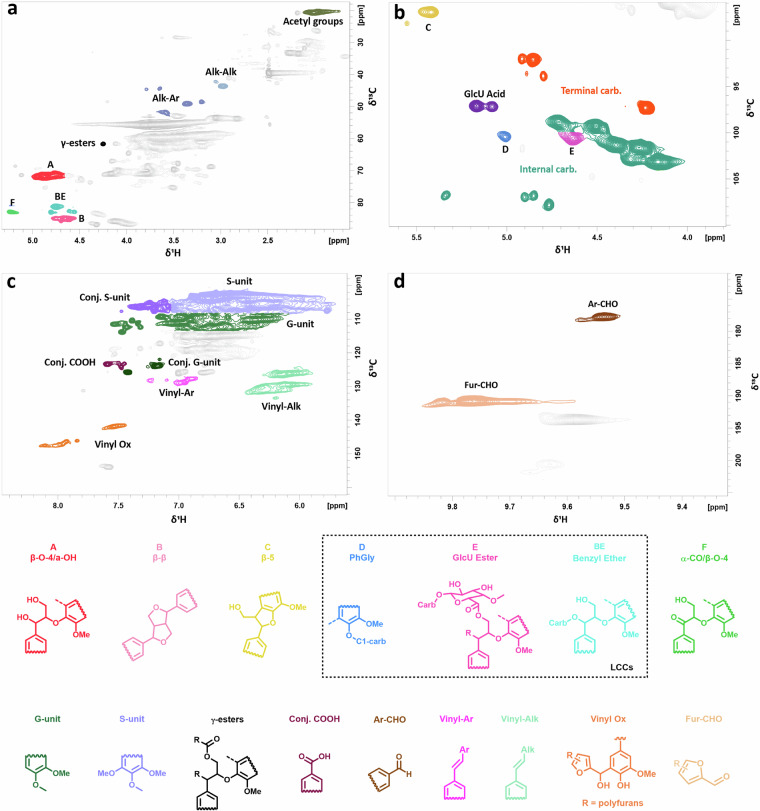
2D HSQC NMR spectra of acetone extracted lignin. The cross-peaks of the main areas of interest were determined according to the corresponding resonances. Each area was colored distinctively to represent the moieties of interest in the oxygenated aliphatic region (**a**), the occurrence of the LCC linkages (**b**) and aromatic region (**c,d**).

One of the primary difficulties in isolating lignin from other biomass components is separating lignin and carbohydrates, largely due to the presence of lignin-carbohydrate complexes (LCCs)^13–16^. In LCCs, lignin and carbohydrates – long or short chain – are chemically bonded mainly through phenyl glycoside, glucuronic ester and benzylic ether linkages (Fig. 2)^14,16–22^. These linkages make LCCs amphiphilic, which gives rise to promising properties for e.g., biomedical applications and surfactants in oil-in-water emulsions^13,23–33^. Although LCCs were initially seen as a significant barrier to the valorization of lignin, they are promising for high value applications. The amphiphilic characteristic of LCCs provides them with good biocompatibility and biological activities such as immunopotentiation, antiviral-, and antioxidant activity^13,23–29^. Additionally, the amphiphilic nature of LCCs enables them to stabilize emulsions and enhance their compatibility with various materials, making them appealing as emulsifiers^30–33^. A major limitation in the valorization of LCCs is that current extraction methods are often complex, time-consuming, and result in low yields^18,34^. To address the need for more efficient protocols, we recently showed that the AquaSolv Omni (AqSO) biorefinery can be optimized to provide a scalable and high-yield route to LCC extraction. AqSO is a green and flexible biorefinery process based on hydrothermal treatment followed by solvent extraction, described by Tarasov *et al*.^17^ and outlined in Fig. 1a for reference.

Machine learning (ML) played a crucial role in the AqSO optimization process, helping to achieve high LCC yields and customize physicochemical properties. ML techniques have been explored to address a wide range of challenges in bio-based materials science^35–39^. For process optimization, which typically requires analyzing a large number of samples^40–43^, Bayesian optimization (BO) has emerged as an alternative to traditional experimental design methods. In BO, an ML model collaborates with a data collection strategy to determine the processing conditions for new sample isolation. These decisions aim to meet specific objectives, such as maximizing yield with minimal sample use.

As part of our previous work, we used BO-guided data collection to curate a set of 90 LCC-containing samples^44^. In this paper, we add 5 additional samples and compile comprehensive measurement data (5 physicochemical properties and NMR spectra) for all 95 LCC samples. To allow for the study of structure-property relationships for multiple properties, we favored a detailed characterization of each sample over sheer number of samples. The resulting Structure-Property LCC (SP-LCC) dataset is bolstered with an extensive technical validation and organized for easy access of the community. For the samples included in SP-LCC, key structural moieties characterized by 2D nuclear magnetic resonance (NMR) spectroscopy and selected physicochemical properties are provided. The measured properties are molar mass distribution, antioxidant activity, glass transition temperature, thermal degradation, and surface tension. We selected these properties, because they are basic materials parameters, but they are also important for certain applications of LCCs, e.g., biomedicine^23,24^, emulsion stabilizers^31,33^ and fillers in thermoplastic formulations^45^. To our knowledge, this is the first time a dataset of this scale is published on lignin, that allows for a comprehensive understanding of the behavior of lignin and LCCs. Furthermore, it provides insight into the structural details and property variations of LCCs based on the isolation process conditions. The significance of SP-LCC extends beyond the AqSO biorefinery because: (1) the correlation between structure and properties holds true regardless of the biorefinery process; (2) general conclusions about the impact of processing conditions on structure and properties can be transferred to other biorefinery models. How transferable our dataset from silver Birch is to other source of materials will have to be clarified in the future with similar datasets. Since all the data presented here was gathered by the same research group, consistency between data points is ensured, making SP-LCC particularly suitable for ML applications. We hope that future ML-driven studies of SP-LCC will reveal the LCC structure-property relationship and ultimately promote the widespread valorization of LCCs tailored for various applications.

## Methods

### Sawdust preparation

We debarked a silver birch (Betula pendula) stem and finely ground it with a Wiley Mill M02 grinder. The obtained sawdust was screened to select a sawdust fraction with a size of 0.5–1.5 mm. The fraction was then exposed to air drying. We removed the extractives from the air-dried sawdust with a Soxhlet apparatus using acetone (99.9% purity) as solvent.

### AquaSolv Omni biorefinery process

As depicted in Fig. 1a), we performed hydrothermal treatment (HTT) on extractive-free sawdust according to the recently reported procedure^17,46^. The resulting lignin structure is heavily affected by the severity of the process, which in AqSO is controlled by the reactor temperature (T), liquid-to-solid ratio (L:S), and residence time. To quantitatively express the severity of the reaction, we combined the reaction temperature and the residence time into the single variable represented as prehydrolysis factor (P-factor). P-factor controls the rate of prehydrolysis in a prehydrolysis-kraft process for a dissolving pulp production that reflects the efficiency of hemicellulose removal from the pulp. It can be found as an area under the curve when plotting the relative reaction rate against time. Relative reaction rate describes the change in the reaction rate at a certain process temperature as opposed to the reference reaction rate at 100 °C (Eq. 1)^1^:

1 $${Ln}\frac{{k}_{H,(T)}}{{k}_{100^\circ {\rm{C}}}}=\frac{{E}_{A,H}}{375.15R}-\frac{{E}_{A,H}}{{RT}}$$

where ${k}_{H,(T)}$ is the rate constant of xylan hydrolysis at the given temperature $t$, and ${E}_{A,H}$ is the activation energy. Consequently, P-factor strongly relies on the activation energy of the fast-reacting xylan. The chosen process variables (P-factor, temperature, and liquid-to-solid ratio) were restricted to the following ranges: 250–1000 for the P-factor, 160–195 °C for the temperature, and 0.25–2 for the liquid-to-solid ratio. P-factor as a single variable is depicted in Eq. 2^1^, where $k$ is the rate constant, $T$ is the reaction temperature (K) and $t$ is the residence time (h). All calculations were carried out according to the assumption that activation energy equals 125.6 kJ mol^−1^.

2 $$P-{factor}={\int }_{0}^{{t}_{f}}\frac{k\left(T(t)\right)}{{k}_{100^\circ {\rm{C}}}}{dt}={\int }_{0}^{{t}_{f}}{e}^{40.48-\frac{15106}{T(t)}}{dt}$$

After the reaction, the resulting solid fraction was exhaustively washed with deionized water and subsequently exposed to acetone (75 vol%, aq.) extraction yielding acetone-extracted lignin (AEL) solutions. LCC-containing AELs were isolated by removing the solvent (75 vol% acetone aq.) through rotary evaporation (*T* = 40 °C, *p* = 20 mbar). Finally, the obtained AELs/LCCs were subjected to vacuum oven drying (*T* = 40 °C, *p* = 5 mbar) under P_2_O_5_ until a constant weight was reached. The dried AELs contained varying amounts of LCC depending on the biorefinery process variables. The obtained samples were labeled to reflect the process conditions used to produce each specific sample and are represented as follows: P840-195-2.00, where P840 is the P-factor employed, 195 is the reaction temperature and 2.00 is the L:S.

### Heteronuclear single-quantum coherence nuclear magnetic resonance

We recorded the 2D NMR using a Bruker AVANCE 600 NMR spectrometer equipped with a CryoProbe^17,46,47^. Approximately 75 mg of each dried LCC-containing sample was dissolved in 0.6 mL of DMSO-d_6_. We defined the acquisition time for the ^1^H-dimension as 77.8 ms with 36 scans per block employing 1024 collected complex points, while 3.94 ms was the set time for the ^13^C-dimension with 256-time increments recorded. We processed the obtained 2D HSQC NMR data using 1024 × 1024 data points and employed the Qsine function for both ^1^H and ^13^C dimensions. To calibrate the chemical shifts, we chose the DMSO peak at δ_C_/δ_H_ 39.5/2.49 ppm/ppm, and we assigned the cross-peaks according to previous reports^14,16,17,46^. The normalized quantification of different lignin and LCC signals was carried out assuming that (Eq. 3)^17,46^:

3 $${\rm{G}}+{\rm{S}}={{\rm{G}}}_{2}+{{\rm{S}}}_{2,6}/2=100{\rm{Ar}}$$

### Molar mass distribution

We determined the molar mass distribution (MMD) with a size exclusion chromatography (SEC) equipped with a multi-angle light scattering (MALS) detector, and a differential refractive index (RI) detector. The MALS detector was equipped with 8 photodiodes with pass filters on every second photodiode. The separation was performed on a Jordi X-stream H_2_O 1000 Å column (10 × 250 mm i.d.) equipped with a guard column (10 × 50 mm i.d.). The column oven was set to 40 °C. We prepared the samples by dissolving 5 mg of each AEL sample in 1 mL of dimethyl sulfoxide (DMSO) containing 0.05 M lithium bromide. We then placed the samples under careful shaking for a longer period to minimize the formation of aggregates. Before the SEC measurements, we filtrated the dissolved AELs over a 0.22 µm nylon syringe filter. We used the following parameters for the molecular weight analyses: 0.5 mL min^−1^ flow rate; 100 µL injection volume; 50 min run time; 0.15 dn/dc value. The obtained data was evaluated using ASTRA 7.3.2. software. We defined the peaks based on the RI concentration curve and selected the photodiodes 2, 4, and 6. The result fitting was adjusted with forward extrapolation. For the MMD we are interested in the following parameters: Number average molecular weight (M_n_), molecular weight at the peak of the distribution curve (M_p_), and average molecular weight (M_w_). Our determination of the MMD was based on the methodology reported by Zinovyev *et al*.^48^.

### Antioxidant properties

For the determination of antioxidant properties of the AELs/LCCs, we used an improved normalized radical scavenging index (nRSI) method^49^. It is based on the standard procedures for antioxidant activity evaluation utilizing 2,2-diphenyl-1-picrylhydrazyl (DPPH) as a reactive free radical^50–52^. Briefly, we prepared a set of LCC/AEL solutions (120–600 mg L^−1^) and a DPPH solution (75 µmol L^−1^) using 90 vol% acetone (aq.) as a solvent in each case. Following that, we mixed the AEL/LCC solutions with the DPPH solution in a lignin:DPPH = 1:39 (v/v) ratio. To measure the change of the DPPH concentration in the prepared solutions, we employed UV-vis spectroscopy using a Shimadzu UV-2550 spectrophotometer and a 10 mm path length quartz cuvette at a wavelength of 515 nm. The absorbance of the solutions was monitored immediately after the preparation and after 24 h when the steady state was reached. In parallel, the absorbance of a LCCs/AEL-free (blank) solution containing 75 µmol L^−1^ DPPH in 90 vol% acetone (aq.) was measured to correct the absorbance values of the AEL/LCC-containing solutions, according to the severity of the DPPH degradation in a given solvent. The lignin absorbance was corrected during the measurements following our recently reported procedure^53^. More details regarding the experimental procedure can be found in the ESI.

### Glass transition temperature

We determined the glass transition temperature (*T*_g_) with modulated differential scanning calorimetry (MDSC, TA Instruments DSC250, Discovery series). We measured approximately 10 mg of AEL in TZero™ aluminum pans under a flow of nitrogen (50 mL min^−1^). The samples were heated from 40 °C to 115 °C at 5 °C min^−1^, followed by cooling to 20 °C. The samples were reheated at 2 °C min^−1^ until reaching 170 °C. We set the modulation amplitude to 1.20 °C and the modulation period to 60 s. We determined *T*_g_ from the last heating ramp as the half-height midpoint of the step-change in the reversible heat flow curve in the TRIOS v5.1.1.5 software.

### Thermal degradation

We studied the thermal stability of the AEL samples by thermal gravimetric analysis (Discovery SDT 650 simultaneous DSC/TGA thermal analyzer, TA Instrument). We measured approximately 7 mg of AEL in an aluminum pan against an empty pan as a reference. The samples were heated to 700 °C at 10 °C min^−1^ under N_2_ atmosphere. The parameters of interest were *T*_onset_ = the start of the thermal degradation, *T*_max_ = temperature where the rate of decomposition reaches its maximum, T_50_ = when 50% of the sample has degraded and the char yield, and we determined these using the TRIOS v5.1.1.5 software. *T*_onset_ and *T*_max_ were determined from a derivative curve of the weight change curve with reference to the temperature curve. The char residue was determined from the weight change curve at maximum temperature.

### Surface tension

We measured the air/water interface of the AELs in aq. NaOH solution (pH 12.65) with a force tensiometer-K100 (Krüss, Germany) using a Wilhelmy plate. In total, we measured five concentrations of each AEL sample (0.5 mg mL^−1^, 0.4 mg mL^−1^, 0.25 mg mL^−1^, 0.1 mg mL^−1^ and 0.08 mg mL^−1^). First, we prepared the 0.5 mg mL^−1^ solution by dissolving 12.5 mg AEL in the aq. NaOH solution in a 25 mL volumetric flask overnight under magnetic stirring. Then we prepared the 0.4 mg mL^−1^ solution by diluting the 0.5 mg mL^−1^ solution directly after being measured. The remaining solutions we prepared in the same way by diluting the previous solution directly after the measurement. Measurement points were taken until 5 measurements with a standard deviation below 0.1 mN m^−1^ were obtained.

### Bayesian optimization

The data collection was guided by BO, building on the approach described by Löfgren *et al*.^35^. A detailed account of the process will be published elsewhere^44^. Briefly, we modeled the AEL and carbohydrate content as independent Gaussian processes using the BOSS code^54^. The models were initialized from a sequence of 12 Sobol points^55^ and the experimental noise was accounted for by incorporating a Gaussian noise, estimated from the technical validation data, into the models. To maintain an optimal workload in the laboratory, we performed the acquisitions in batches of 8 data points each using a Kriging-believer approach^56^. The first five batches contained two exploration-modified LCB acquisitions^57,58^ each for the AEL and carbohydrates, and two exploratory acquisitions where only the standard deviation of the models was minimized. To obtain the optimal workload of eight samples, we added two test data points to these five batches, generated independently from a continuation of the initial Sobol sequence. In the last two batches, these test points were dropped in favor of two additional exploratory acquisitions. In total, we collected 54 data points, including initial points, over seven batches. Furthermore, we collected 14 test points, of which four were collected outside of the seven batches. We used these test points for model validation and to terminate the BO once the prediction errors dropped below 10% relative to the observed measurement range.

## Data Records

Our database comprises 95 samples isolated under 72 different process conditions. We characterized 88 of the samples with 2D HSQC NMR. Among the characterized samples, the lignin yield was too low for 13 samples to measure any of the physicochemical properties. To bolster the available property information on low-yield samples, we re-isolated seven such samples. For these samples, we decided to forgo measuring the 2D HSQC NMR characterization in favor of measuring all the properties. We refer to these seven samples as replicates, and these are distinguished by an “-R” added to their sample ID. To validate the biorefinery reproducibility, we isolated four samples under identical process conditions. We included these in the SP-LCC dataset and distinguished them by adding “NMRValidation” in their sample ID. No physicochemical properties were measured for these samples.

The SP-LCC dataset is available on Figshare^59^. It is structured in the following way:

- The sample ID, processing condition, yield, lignin moieties identified from 2D HSQC NMR, and properties for all samples are available in the CSV file named “SP-LCC_table.csv”. The file is structured as described in Table 1.

**Table 1:** Description of the scalar data in the SP-LCC dataset and contained in the CSV file “SP-LCC_table.csv”.

Column name	Description	Range	Nr. of entries	Unit
SampleID	A unique ID for each sample.	not applicable	95	—
P-factor	The severity of the HTT.	(250,1000)	95	—
Temperature	The temperature of the HTT.	(160,195)	95	°C
L:S	The liquid-to-solid ratio in the HTT.	(0.25,2.00)	95	—
Yield	The dry yield of AEL samples.	(0.48,19.80)	95	%
Carbohydrate_content -Ar-CHO	The following columns describe lignin moieties evaluated and calculated from 2D HSQC NMR^17,46^. The structures of the moieties are depicted in Fig. 2.	—	88	per 100 Ar
Mn_1-Mn_4	The number average molecular weight (Mn) of AEL samples.	(3.60,11.50)	58	kDa
Mp_1-Mp_4	The molecular weight at the peak of the distribution curve (M_p_) of AEL samples.	(3.60,8.40)	58	kDa
Mw_1-Mw_4	The weight average molecular weight (M_W_) of AEL samples.	(4.60,117.00)	58	kDa
nRSI_1-nRSI_4	Normalized radical scavenging index of AEL samples.	(3.64,8.59)	73	mmol g^−1^
Tg_1-Tg_4	Glass transition temperature of AEL samples.	(87.73,133.82)	59	°C
T-onset_1-T-onset_4	The temperature when thermal degradation of the AEL samples initiates.	(148.47,197.38)	59	°C
T-max_1-T-max_4	The temperature where the rate of decomposition reaches its maximum.	(344.14,374.50)	59	°C
T-50_1-T-50_4	The temperature at which 50% of the sample has thermally degraded.	(361.40,447.34)	59	°C
Char_yield_1-Char_yield_4	Describes how much of the weight remains by the end of the heating at 700 °C.	(26.89,39.19)	59	%
ST-0.5_1-ST-0.5_5	The surface tension of AEL samples at 0.5 mg mL^−1^ concentration.	(51.83,64.19)	57	mN m^−1^
ST-0.4_1-ST-0.4_5	The surface tension of AEL samples at 0.4 mg mL^−1^ concentration.	(55.05,65.53)	57	mN m^−1^
ST-0.25_1-ST-0.25_5	The surface tension of AEL samples at 0.25 mg mL^−1^ concentration.	(57.85,67.20)	57	mN m^−1^
ST-0.1_1-ST-0.1_5	The surface tension of AEL samples at 0.1 mg mL^−1^ concentration.	(59.91,69.84)	57	mN m^−1^
ST-0.08_1-0.08_5	The surface tension of AEL samples at 0.08 mg mL^−1^ concentration.	(61.24,70.37)	57	mN m^−1^
- The intensities of the NMR spectra are contained in the archive “NMR_spectra.zip”, which contains one file for the intensities per sample named “NMRspectrum_{sampleID}.csv”. The corresponding chemical shifts, at which these intensities were measured, are contained in the same archive NMR_spectra.zip in two files per sample, for the x-axis (F1 C axis) “xarr_{sampleID}.csv” and the y-axis (F2 H axis) “yarr_{sampleID}.csv”, where {sampleID} refers to the sample ID of the respective sample. The files are structured as described in Table 2.

**Table 2:** Description of the non-scalar data comprised in the presented SP-LCC dataset.

File name	Description	Range	Nr. of entries	Unit
NMR_spectrum_{sampleID}.csv	Contains the intensities of the NMR spectra. Rows correspond to the y-axis and columns to the x-axis.	Different for each sample	1024 rows × 1024 columns	Arbitrary units
xarr_{sampleID}.csv	Contains the chemical shifts of the x-axis (F1 C axis)	(−3.1, 211.8) is slightly shifted for each sample	1024	Parts per million
yarr_{sampleID}.csv	Contains the chemical shifts of the y-axis (F2 H axis)	(−0.25, 10.71) is slightly shifted for each sample	1024	Parts per million
RI_Chromatogram_{sampleID}.csv	Contains the retention time (min) and the refractive index (dRI)	Varies between samples	Varies between samples	min dRI
LS_Chromatogram_{sampleID}.csv	Contains the retention time (min) and light scattering (Rayleigh ratio)	Varies between samples	Varies between samples	min Rayleigh ratio
Tg_Thermogram_{sampleID}.csv	Column 1: Time Column 2: Temperature Column 3: Heat flow Column 4: Reversing heat flow Non-reversing heat flow	Varies between samples	Varies between samples	min °C W g^−1^ W g^−1^
TD_Thermogram_{sampleID}.csv	Column 1: Time Column 2: Temperature range Column 3: Heat flow Column 4: Weight change of sample Column 5: Weight derivative against temperature	Varies between samples	Varies between samples	min °C W g^−1^% % °C^−1^

There are multiple files per sample comprising all the measurement data.
- The CSV files containing the NMR data were generated from Bruker files, which we make available, too, in the “NMR_spectra_Bruker.zip” archive. In the archive, every subfolder is named by the sample ID of the respective sample.
- For full transparency, we also include python scripts to parse and plot the NMR spectra both from the Bruker files, with “parse_and_plot_Bruker_NMR_spectrum.py”, and from the.csv files with “plot_NMR_spectrum.py”. The dependencies for their use are described in the file “readme.txt”.
- The chromatograms are contained in the archives “RI_Chromatogram.zip” and “LS_Chromatogram.zip” with one file per sample named “RI_Chromatogram_{sampleID}.csv” the refractive index and “LS_Chromatogram_{sampleID}.csv” for light scattering chromatograms. For parallel measurements, “_2” has been added to the file name. The files are structured as described in Table 2. Additional details of the chromatogram data are described in section 2 of the Supplementary information.
- The thermograms are contained in the archives “TD_Thermogram.zip” and “Tg_Thermogram.zip” with one file per sample named “Tg_Thermogram_{sampleID}.csv” for thermograms obtained from DSC analyses and “TD_Thermogram_{sampleID}.csv” obtained from TGA analyses. For parallel measurements, “_2” has been added to the file name. The files are structured as described in Table 2. Additional details of the thermogram data are described in section 2 of the Supplementary information.

## Technical Validation

For the technical validation, we consider three sources of uncertainty, as shown in Fig. 1b): (I) uncertainty related to sample preparation, i.e., the degree of reproducibility of the biorefinery process; (II) measurement uncertainty related to NMR measurements; (III) measurement uncertainty related to the physicochemical properties of lignin, i.e., from MMD, antioxidant activity, *T*_g_, surface tension, and thermal degradation measurements.

### Evaluation of the biorefinery process reproducibility

To evaluate the reproducibility of the biorefinery process, we investigated four replicates generated for identical processing conditions: P-factor = 500; L:S = 1; T = 195 °C (See Table 1 in section 3 of the Supplementary information). The corresponding spectra in the dataset contain “NMRValidation” as suffix in their ID.

We compared the structure, as determined by HSQC NMR (Fig. 2), and the biorefinery yield of the replicates. For this analysis, we considered the structure rather than the physicochemical properties for two reasons: (1) the structure of LCCs influences their properties; (2) in contrast to the property measurements, the noise of NMR measurements is small, as seen in subsection (II). This allowed us to conveniently isolate the uncertainty derived from the biorefinery process.

To quantify the deviation in lignin structure, we calculated the standard deviation (SD) and relative standard deviation (RSD) of the identified lignin moieties (Table 2). The RSD is reported relative to the average of the four measured samples. Repeated tests indicate that the outcome of the experiments was very similar in all cases with an average RSD of 5% (Table 3, entry 26), which is consistent with previous studies^17,46,47,60^. Notably, for the structural characterization, the observed SD and RSD depend on the moiety under consideration. In particular, the quantification of low concentration lignin units (<1 mol%) is heavily affected by the background noise^60^. In general, we note that the deviation between replicates depends on the processing conditions. For four moieties of interest (β-O-4, BE, β-β, and carbohydrates), the SD is visualized in Fig. S1 in the Supplementary information (section 4).

**Table 3:** The standard deviation and relative standard deviation of the experimental errors in the NMR measurements for different lignin moieties.

Entry	Lignin moiety	Uncertainty from the biorefinery process (I)		Uncertainty from NMR measurement (II)	
		Standard deviation (per 100 Ar)	Relative standard deviation (%)	Standard deviation (per 100 Ar)	Relative standard deviation (%)
1	Syringyl/Guaiacyl ratio	0.02	0.78	0.02	0.7
2	Acetyl groups	0.24	3.92	0.08	0.9
3	Alkyl-Alkyl	0.06	3.69	0.09	2.3
4	Alkyl-Aryl	0.21	10.44	0.85	7.51
5	γ-esters	0.01	1.06	0.12	20.81
6	β-O-4/α-OH	0.62	2.96	0.03	1.84
7	BE total	0.10	3.08	0.01	0.25
8	α-CO/β-O-4	0.02	1.87	0.04	3.54
9	Resinol (β-β)	0.03	0.51	0.05	0.75
10	Phenylcoumaran (β-5)	0.05	2.02	0.03	1.20
11	GlcU Acid	0.03	5.14	0.01	5.67
12	GlcU Esters	0.02	4.24	0.00	0.00
13	PhGly	0.03	8.21	0.00	0.00
14	Terminal carbohydrate	0.12	5.50	0.03	1.60
15	Internal carbohydrate	0.61	5.27	0.06	1.45
16	Total carbohydrate^a^	0.71	5.20	0.09	1.50
17	Carbohydrate DP^b^	0.22	3.42	0.00	0.10
18	Conjugated Syringyl	0.76	7.05	0.28	1.78
19	Conjugated COOH	0.05	4.65	0.01	0.70
20	Conjugated Guaiacyl	0.10	2.47	0.02	0.48
21	Vinyl-Ar	0.69	3.56	0.06	4.02
22	Vinyl-Alk	0.15	2.28	0.12	1.12
23	Vinyl Ox	0.01	0.72	0.06	3.17
24	Fur-CHO	0.02	11.11	0.01	2.54
25	Ar-CHO	0.24	16.42	0.04	1.08
26	Average RSD	4.62		2.60	

The experiments were repeated for four samples isolated under the same conditions (P-factor = 500, L:S = 1, T = 195 °C, Sawdust dry matter = 4 g). Consecutive measurements describe the experimental errors of two consecutive measurements of the NMR tube with the sample obtained at P-factor = 625, L:S = 1.13 and T = 178 °C.

^a^Internal + terminal carbohydrates. ^b^Average carbohydrate chain length calculated as the ratio between total and terminal carbohydrates^17,46^. SD = standard deviation. RSD = relative standard deviation.

The four samples used for validating the quantification of HSQC NMR were also used for evaluating the yield variability across replicates. The corresponding SD and the RSD are reported in Table 3, and their calculations are presented in Table S1 in the Supplementary Information. The RSD of the AEL yield is 4%.

### Measurement uncertainty related to the HSQC NMR analysis

To quantify the NMR uncertainty, we performed two consecutive HSQC measurements on a single LCC sample obtained at P-factor = 625, L:S = 1.13, and T = 178 °C. The sample was first dissolved in DMSO-d_6_ and then transferred to an NMR tube. As soon as the first measurement was completed, the sample was removed from the magnet, placed back into the magnet and the second HSQC measurement was recorded on the same tube. We employed the same HSQC conditions used throughout this work (see the Methods section).

The average RSD (Table 3) for the quantified lignin moieties between the two measurements was 2.6%, signifying a low error related to the HSQC NMR analysis.

### Measurement uncertainty related to the physicochemical properties of lignin

**Fig. 3:**
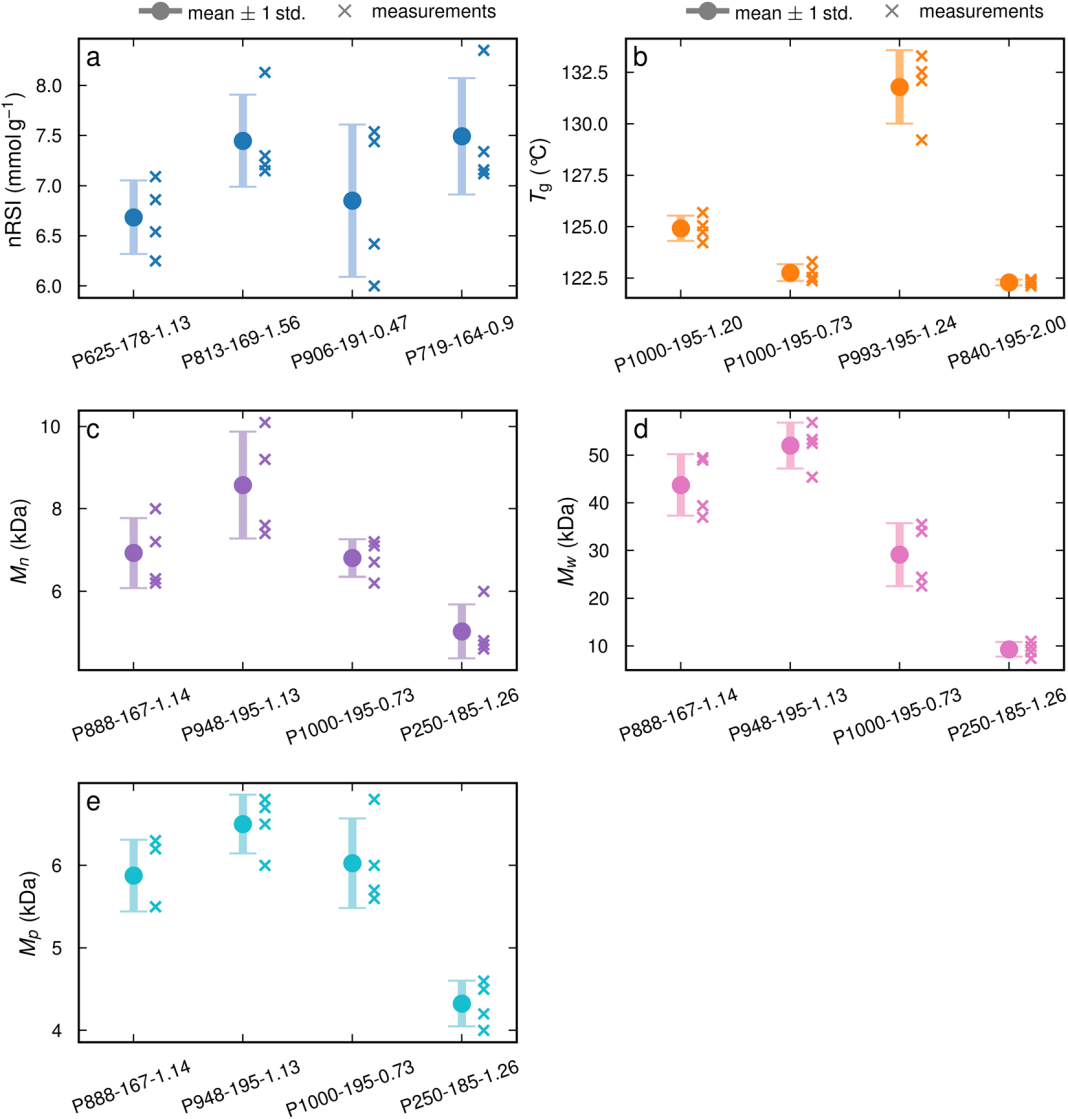
Measurement uncertainties for the (**a**) Normalized radical scavenging index, (**b**) glass-transition temperature, (**c**) number average molecular weight, (**d**) average molecular weight, and (**e**) molecular weight at the peak of the distribution curve. The error bars indicate the mean standard deviation and were calculated from 4 repeated measurements (crosses) for each of the four samples (x-axis) produced under different processing conditions.

**Fig. 4:**
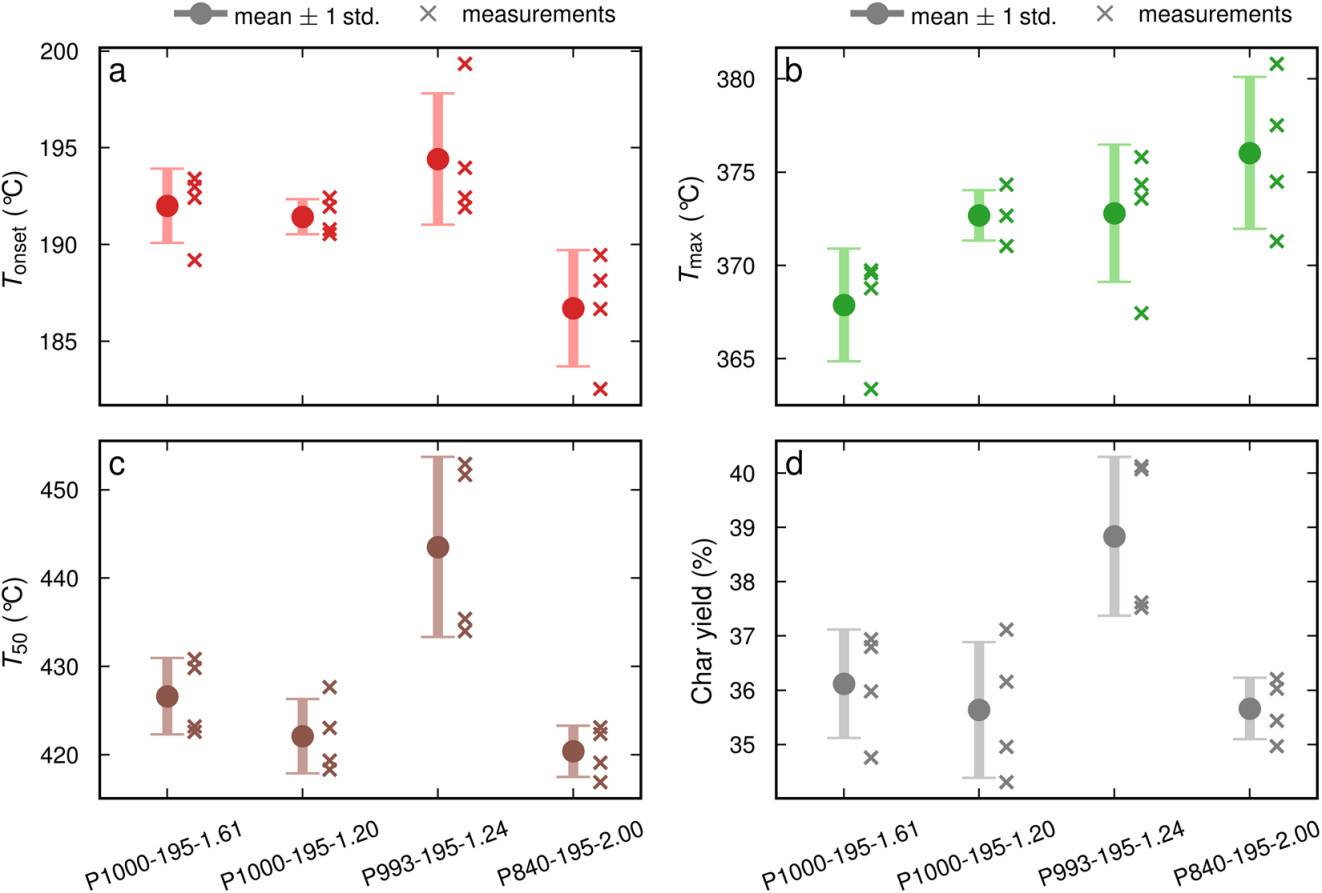
Measurement uncertainties for properties related to thermal degradation: (**a**) onset temperature, (**b**) temperature at maximum degradation, (**c**) temperature after degradation of 50% material weight, and (**d**) char yield. The error bars indicate the mean standard deviation, calculated from 4 repeated measurements (crosses) for each of the four samples (x-axis) produced under different processing conditions.

**Fig. 5:**
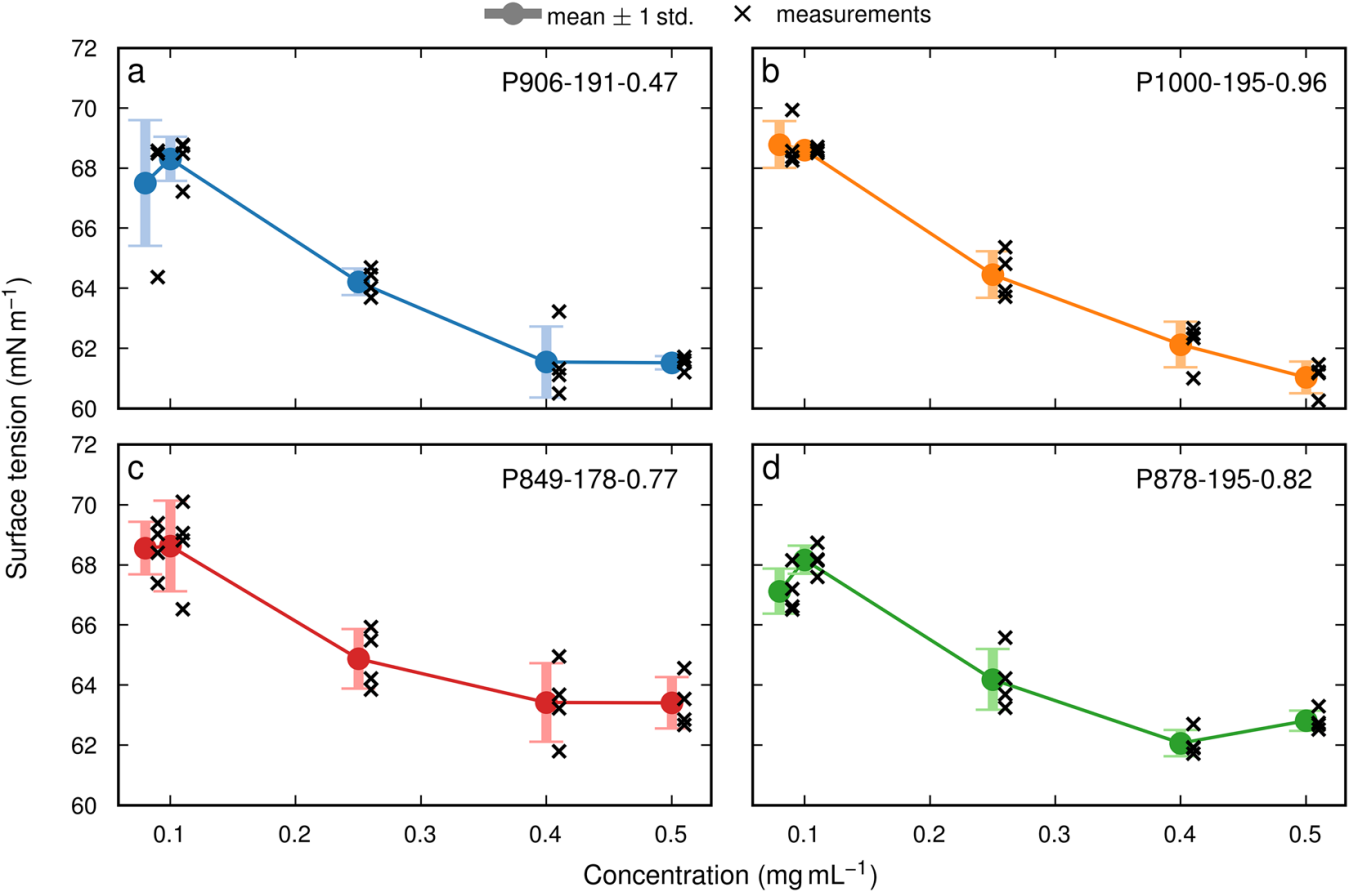
Measurement uncertainties for the surface tension (y-axis) at four different concentrations (x-axis). The error bars indicate the mean standard deviation and were calculated from 4 repeated measurements (crosses) at each concentration level, for each of the four samples produced under different processing conditions.

The technical validation for MMD, nRSI, *T*_g_, and thermal degradation was carried out by repeating measurements four times for four selected AEL samples. For the surface tension, a total of five parallel measurements were performed, however, only four were selected for technical validation as the fifth was discarded as an outlier (see Fig. S2 in section 5 of the Supplementary information). Table 4 contains the average SD and the average RSD (relative to the mean of measured properties) across all four validation samples. The calculations are described in Table S2 and Table S3 in the Supplementary information (section 6). In Figs. 3–5 the standard deviation of each individual sample is shown.

**Table 4:** The average standard deviation and average relative standard deviation of selected properties were measured using four parallel measurements on four AEL samples.

Type of property	Average standard deviation	Average relative standard deviation
Yield	0.51%	3.85%
Molar mass distribution:		
Number average molecular weight (M_n_)	0.87 kDa	11.77%
Molecular weight at the peak of the distribution curve (M_p_)	0.41 kDa	7.07%
Average molecular weight (M_w_)	5.25 kDa	15.79%
Normalized radical scavenging index	0.56 mmol g^−1^	7.62%
Glass transition temperature	0.97 °C	0.57%
Thermal degradation:		
*T*_onset_	2.50 °C	1.21%
*T*_max_	3.20 °C	0.81%
*T*_50_	6.10 °C	1.25%
Char yield	1.12%	2.91%
Surface tension	0.93 mN m^−1^	1.24%

As shown in Table 4, the RSD of the repeated measurements varies between physiochemical properties. For most properties, the RSD is low with values well below 5%. The RSDs of the nRSI with 7.62% and the MMD parameters with up to 15.79% are the exceptions. The high RSD of the MMD parameters is most likely due to the formation of aggregates during the preparation of the AEL-DMSO/LiBr solutions for the MMD measurements^61^. The aggregates are present in low concentrations; however, they have a high impact on light scattering and will therefore be detected by the MALS detector^62^. The M_W_ and M_n_ are calculated based on the averages of all detected molecular weights in the sample, which means the aggregates will affect these parameters. By contrast, the M_p_ gives the molecular weight at the highest detected concentration. In this molecular weight fraction, the aggregates are not present, and the measurement uncertainty associated with M_p_ is, therefore, lower than for the M_n_ and M_W_.

Comparing the SD of the measured properties with the literature was not possible, as such thorough validation of measurement performed on lignin is typically not performed or not reported. For all properties, including those that vary more strongly with repeated experiments, the SD is considerably lower than the range of observed values across samples. Thus, all measured property data can be meaningfully interpreted within our dataset. We report the range of the measured property values and compare them with the literature in Table S4 in the Supplementary Information.

We also carried out technical validation for the chromatograms and thermograms obtained from the MMD, *T*_g_, and thermal degradation measurements. We did this by extracting the data from the four measurements performed for each AEL sample. The point-wise SD of the chromatograms and thermograms of all four measurements was calculated based on the extracted CSV files and is reported in Fig. S3-6 in the Supplementary information (section 8). The validation of the most important points of the chromatograms and thermograms have been done in combination with the technical validation of their corresponding physicochemical properties and has been described above.

## Supplementary information

SP-LCC — Supplementary Information

## Data Availability

The code used to guide and analyze the data collection is based on the BOSS python library for Bayesian optimization and is distributed as a GitLab repository (https://gitlab.com/cest-group/lcc_biorefinery_optimization). The repository includes instructions for installation, including version requirements, and usage.

## References

[1] Sixta, H. Handbook of Pulp; Wiley-VCH Verlag GmbH & Co. KGaA, Weinheim, (2006).

[2] Gillet, S. *et al*. Lignin Transformations for High Value Applications: Towards Targeted Modifications Using Green Chemistry. *Green Chem.* **19**, 4200–4233, 10.1039/C7GC01479A (2017).

[3] Cao, L. *et al*. Lignin Valorization for the Production of Renewable Chemicals: State-of-the-Art Review and Future Prospects. *Bioresour. Technol.* **269**, 465–475, 10.1016/j.biortech.2018.08.065 (2018). 30146182 10.1016/j.biortech.2018.08.065

[4] Berlin, A., Balakshin, M. Chapter 18-Industrial Lignins: Analysis, Properties, and Applications, in *Bioenergy Research: Advances and Applications*. (eds. Gupta, V. K., Tuohy, M. G., Kubicek, C. P., Sadler, J., Xu, F.) 315–336, 10.1016/B978-0-444-59561-4.00018-8 (Elsevier, 2014).

[5] Obydenkova, S. V., Kouris, P. D., Hensen, E. J. M., Heeres, H. J. & Boot, M. D. Environmental Economics of Lignin Derived Transport Fuels. *Bioresour. Technol.* **243**, 589–599, 10.1016/j.biortech.2017.06.157 (2017). 28709064 10.1016/j.biortech.2017.06.157

[6] Obydenkova, S. V. *et al*. Industrial Lignin from 2G Biorefineries – Assessment of Availability and Pricing Strategies. *Bioresour. Technol.* **291**, 121805, 10.1016/j.biortech.2019.121805 (2019). 31351376 10.1016/j.biortech.2019.121805

[7] Dessbesell, L., Paleologou, M., Leitch, M., Pulkki, R. & Xu, C. Global Lignin Supply Overview and Kraft Lignin Potential as an Alternative for Petroleum-Based Polymers. *Renewable and Sustainable Energy Rev.* **123**, 109768, 10.1016/j.rser.2020.109768 (2020).

[8] Ragauskas, A. J. *et al*. Lignin Valorization: Improving Lignin Processing in the Biorefinery. *Science.* **344**, 1246843–1246843, 10.1126/science.1246843 (2014). 24833396 10.1126/science.1246843

[9] Tuck, C. O., Pérez, E., Horváth, I. T., Sheldon, R. A. & Poliakoff, M. Valorization of Biomass: Deriving More Value from Waste. *Science.* **337**, 695–699, 10.1126/science.1218930 (2012). 22879509 10.1126/science.1218930

[10] Argyropoulos, D. D. S. *et al*. Kraft Lignin: A Valuable, Sustainable Resource, Opportunities and Challenges. *ChemSusChem.* **16**, e202300492, 10.1002/cssc.202300492 (2023). 37493340 10.1002/cssc.202300492

[11] Ralph, J. *et al*. Lignins: Natural Polymers from Oxidative Coupling of 4-Hydroxyphenyl- Propanoids. *Phytochem. Rev.* **3**, 29–60, 10.1023/B:PHYT.0000047809.65444.a4 (2004).

[12] Vanholme, R., Demedts, B., Morreel, K., Ralph, J. & Boerjan, W. Lignin Biosynthesis and Structure. *Plant Physiol.* **153**, 895–905, 10.1104/pp.110.155119 (2010). 20472751 10.1104/pp.110.155119 PMC2899938

[13] Tarasov, D., Leitch, M. & Fatehi, P. Lignin–Carbohydrate Complexes: Properties, Applications, Analyses, and Methods of Extraction: A Review. *Biotechnol. Biofuels.* **11**, 1–28, 10.1186/s13068-018-1262-1 (2018). 30288174 10.1186/s13068-018-1262-1 PMC6162904

[14] Balakshin, M. Y., Capanema, E. A. & Chang, H. M. MWL Fraction with a High Concentration of Lignin-Carbohydrate Linkages: Isolation and 2D NMR Spectroscopic Analysis. *Holzforschung.* **61**, 1–7, 10.1515/HF.2007.001 (2007).

[15] Lawoko, M., Henriksson, G. & Gellerstedt, G. Characterisation of Lignin-Carbohydrate Complexes (LCCs) of Spruce Wood (Picea Abies L.) Isolated with Two Methods. *Holzforschung.* **60**, 156–161, 10.1515/HF.2006.025 (2006).

[16] Balakshin, M., Capanema, E., Gracz, H., Chang, H.-min & Jameel, H. Quantification of Lignin-Carbohydrate Linkages with High-Resolution NMR Spectroscopy. *Planta.* **233**, 1097–1110, 10.1007/s00425-011-1359-2 (2011). 21298285 10.1007/s00425-011-1359-2

[17] Tarasov, D. *et al*. AqSO Biorefinery: A Green and Parameter-Controlled Process for the Production of Lignin–Carbohydrate Hybrid Materials. *Green Chem.* **24**, 6639–6656, 10.1039/D2GC02171D (2022).

[18] Balakshin, M., Capanema, E., Berlin, A. Chapter 4-Isolation and Analysis of Lignin–Carbohydrate Complexes Preparations with Traditional and Advanced Methods: A Review in *Studies in Natural Products Chemistry* Vol. 42 (ed. Atta-ur-Rahman) 83–115, 10.1016/B978-0-444-63281-4.00004-5 (Elsevier, 2014).

[19] Giummarella, N., Zhang, L., Henriksson, G. & Lawoko, M. Structural Features of Mildly Fractionated Lignin Carbohydrate Complexes (LCC) from Spruce. *RSC Adv.* **6**, 42120–42131, 10.1039/C6RA02399A (2016).

[20] Giumarella, N. & Lawoko, M. Structural Insights on Recalcitrance during Hydrothermal Hemicellulose Extraction from Wood. *ACS Sustain. Chem. Eng.* **5**, 5156–5165, 10.1021/acssuschemeng.7b00511 (2017).

[21] Capanema, E., Balakshin, M., Katahira, R., Chang, H. M. & Jameel, H. How Well Do MWL and CEL Preparations Represent the Whole Hardwood Lignin? *J. Wood Chem. Technol.* **35**, 17–26, 10.1080/02773813.2014.892993 (2015).

[22] Nishimura, H., Kamiya, A., Nagata, T., Katahira, M. & Watanabe, T. Direct Evidence for α Ether Linkage between Lignin and Carbohydrates in Wood Cell Walls. *Sci. Rep.* **8**, 1–11, 10.1038/s41598-018-24328-9 (2018). 29695732 10.1038/s41598-018-24328-9 PMC5916878

[23] Sakagami, H. *et al*. Molecular Requirements of Lignin–Carbohydrate Complexes for Expression of Unique Biological Activities. *Phytochemistry.* **66**, 2108–2120, 10.1016/j.phytochem.2005.05.013 (2005). 15978643 10.1016/j.phytochem.2005.05.013

[24] Sakagami, H., Kushida, T., Oizumi, T., Nakashima, H. & Makino, T. Distribution of Lignin–Carbohydrate Complex in Plant Kingdom and Its Functionality as Alternative Medicine. *Pharmacol. Ther.* **128**, 91–105, 10.1016/j.pharmthera.2010.05.004 (2010). 20547183 10.1016/j.pharmthera.2010.05.004

[25] Huang, C. *et al*. Unveiling the Structural Properties of Lignin-Carbohydrate Complexes in Bamboo Residues and Its Functionality as Antioxidants and Immunostimulants. *ACS Sustain. Chem. Eng*. 2018, **6**, 12522–12531, 10.1021/acssuschemeng.8b03262 (2018).

[26] Jiang, B. *et al*. Structure-Antioxidant Activity Relationship of Active Oxygen Catalytic Lignin and Lignin-Carbohydrate Complex. *Int. J. Biol. Macromol.* **139**, 21–29, 10.1016/j.ijbiomac.2019.07.134 (2019). 31374268 10.1016/j.ijbiomac.2019.07.134

[27] Xie, D. *et al*. Structural Characterization and Antioxidant Activity of Water-Soluble Lignin-Carbohydrate Complexes (LCCs) Isolated from Wheat Straw. *Int. J. Biol. Macromol.* **161**, 315–324, 10.1016/j.ijbiomac.2020.06.049 (2020). 32531357 10.1016/j.ijbiomac.2020.06.049

[28] Pei, W. *et al*. Isolation and Identification of a Novel Anti-Protein Aggregation Activity of Lignin-Carbohydrate Complex From Chionanthus Retusus Leaves. *Front. Bioeng. Biotechnol.* **8**, 573991, 10.3389/fbioe.2020.573991 (2020). 33102457 10.3389/fbioe.2020.573991 PMC7546364

[29] Dong, H. *et al*. Characterization and Application of Lignin-Carbohydrate Complexes from Lignocellulosic Materials as Antioxidants for Scavenging *in Vitro* and *in Vivo* Reactive Oxygen Species. *ACS Sustain. Chem. Eng.* **8**, 256–266, 10.1021/acssuschemeng.9b05290 (2020).

[30] Lahtinen, M. H. *et al*. Lignin-Rich PHWE Hemicellulose Extracts Responsible for Extended Emulsion Stabilization. *Front. Chem.* **7**, 489961, 10.3389/fchem.2019.00871 (2019). 10.3389/fchem.2019.00871 PMC6927942 31921786

[31] Carvalho, D. M. D., Lahtinen, M. H., Bhattarai, M., Lawoko, M. & Mikkonen, K. S. Active Role of Lignin in Anchoring Wood-Based Stabilizers to the Emulsion Interface. *Green Chem.* **23**, 9084–9098, 10.1039/D1GC02891J (2021).

[32] Li, Y. F. *et al*. Comparison of Emulsifying Capacity of Two Hemicelluloses from Moso Bamboo in Soy Oil-in-Water Emulsions. *RSC Adv.* **10**, 4657–4663, 10.1039/C9RA08636F (2020). 35495257 10.1039/c9ra08636f PMC9049161

[33] Lehtonen, M. *et al*. Phenolic Residues in Spruce Galactoglucomannans Improve Stabilization of Oil-in-Water Emulsions. *J. Colloid Interface Sci.* **512**, 536–547, 10.1016/J.JCIS.2017.10.097 (2018). 29100158 10.1016/j.jcis.2017.10.097

[34] Giummarella, N., Pu, Y., Ragauskas, A. J. & Lawoko, M. A Critical Review on the Analysis of Lignin Carbohydrate Bonds. *Green Chem.* **21**, 1573–1595, 10.1039/C8GC03606C (2019).

[35] Löfgren, J. *et al*. Machine Learning Optimization of Lignin Properties in Green Biorefineries. *ACS Sustain. Chem. Eng.* **10**, 9469–9479, 10.1021/acssuschemeng.2c01895 (2022).

[36] Liao, M. & Yao, Y. Applications of Artificial Intelligence-Based Modeling for Bioenergy Systems: A Review. *GCB Bioenergy.* **13**, 774–802, 10.1111/gcbb.12816 (2021).

[37] Velidandi, A. *et al*. State-of-the-Art and Future Directions of Machine Learning for Biomass Characterization and for Sustainable Biorefinery. *J. Energy Chem.* **81**, 42–63, 10.1016/j.jechem.2023.02.020 (2023).

[38] Sharma, V. *et al*. Di. Advances in Machine Learning Technology for Sustainable Biofuel Production Systems in Lignocellulosic Biorefineries. *Sci. Total Environ.* **886**, 163972, 10.1016/j.scitotenv.2023.163972 (2023). 37164089 10.1016/j.scitotenv.2023.163972

[39] Ge, H., Zheng, J. & Xu, H. Advances in Machine Learning for High Value-Added Applications of Lignocellulosic Biomass. *Bioresour. Technol.* **369**, 128481, 10.1016/j.biortech.2022.128481 (2023). 36513310 10.1016/j.biortech.2022.128481

[40] Pham, V. & El-Halwagi, M. Process Synthesis and Optimization of Biorefinery Configurations. *AIChE Journal.* **58**, 1212–1221, 10.1002/aic.12640 (2012).

[41] Maity, S. K. Opportunities, Recent Trends and Challenges of Integrated Biorefinery: Part I. *Renewable and Sustainable Energy Rev.* **43**, 1427–1445, 10.1016/j.rser.2014.11.092 (2015).

[42] Espinoza Pérez, A. T., Camargo, M., Narváez Rincón, P. C. & Alfaro Marchant, M. Key Challenges and Requirements for Sustainable and Industrialized Biorefinery Supply Chain Design and Management: A Bibliographic Analysis. *Renewable and Sustainable Energy Rev.* **69**, 350–359, 10.1016/j.rser.2016.11.084 (2017).

[43] Singh, N. *et al*. Global Status of Lignocellulosic Biorefinery: Challenges and Perspectives. *Bioresour. Technol.* **344**, 126415, 10.1016/j.biortech.2021.126415 (2022). 34838977 10.1016/j.biortech.2021.126415

[44] Diment, D. *et al*. Enhancing Lignin-Carbohydrate Complexes Production and Properties With Machine Learning. *ChemSusChem*. e202401711, 10.1002/cssc.202401711 (2024). 10.1002/cssc.202401711 PMC11997930 39585801

[45] Sen, S., Patil, S. & Argyropoulos, D. S. Thermal Properties of Lignin in Copolymers, Blends, and Composites: A Review. *Green Chem.* **17**, 4862–4887, 10.1039/C5GC01066G (2015).

[46] Schlee, P., Tarasov, D., Rigo, D. & Balakshin, M. Advanced NMR Characterization of Aquasolv Omni (AqSO) Biorefinery Lignins/Lignin-Carbohydrate Complexes. *ChemSusChem.* **16**, e202300549, 10.1002/cssc.202300549 (2023). 37218461 10.1002/cssc.202300549

[47] Rigo, D. *et al*. Upgrading AquaSolv Omni (AqSO) Biorefinery: Access to Highly Ethoxylated Lignins in High Yields through Reactive Extraction (REx). *Green Chem.* **26**, 2623–2637, 10.1039/D3GC03776B (2024).

[48] Zinovyev, G. *et al*. Getting Closer to Absolute Molar Masses of Technical Lignins. *ChemSusChem.* **11**(18), 3259–3268, 10.1002/cssc.201801177 (2018). 29989331 10.1002/cssc.201801177 PMC6175078

[49] Diment, D. *et al*. Study toward a More Reliable Approach to Elucidate the Lignin Structure-Property-Performance Correlation. *Biomacromolecules.*** 25**(1) 200-212, 10.1021/acs.biomac.3c00906 (2024). 10.1021/acs.biomac.3c00906 PMC10777350 38112036

[50] Brand-Williams, W., Cuvelier, M. E. & Berset, C. Use of a Free Radical Method to Evaluate Antioxidant Activity. *LWT-Food Science and Technology.* **28**, 25–30, 10.1016/S0023-6438(95)80008-5 (1995).

[51] Dizhbite, T., Telysheva, G., Jurkjane, V., Viesturs, U. Characterization of the Radical Scavenging Activity of Lignins - Natural Antioxidants. *Bioresour. Technol*. 2004, **95**, 309–317, 10.1016/j.biortech.2004.02.024 (2004). 10.1016/j.biortech.2004.02.024 15288274

[52] Ponomarenko, J. *et al*. Antioxidant Activity of Various Lignins and Lignin-Related Phenylpropanoid Units with High and Low Molecular Weight. *Holzforschung.* **69**, 795–805, 10.1515/hf-2014-0280 (2015).

[53] Diment, D., Musl, O., Balakshin, M., Rigo, D. Guidelines for Evaluating the Antioxidant Activity of Lignin via the 2,2-Diphenyl-1-Picrylhydrazyl (DPPH) Assay. *ChemSusChem*. e202402383, 10.1002/CSSC.202402383 (2025). 10.1002/cssc.202402383 40105287

[54] Todorović, M., Gutmann, M. U., Corander, J. & Rinke, P. Bayesian Inference of Atomistic Structure in Functional Materials. *npj Comput. Mater.* **5**, 1–7, 10.1038/s41524-019-0175-2 (2019).

[55] Sobol’, I. M. On the Distribution of Points in a Cube and the Approximate Evaluation of Integrals. *USSR Computational Mathematics and Mathematical Physics.* **7**, 86–112, 10.1016/0041-5553(67)90144-9 (1967).

[56] Ginsbourger, D., Le Riche, R., Carraro, L. Chapter 6-Kriging Is Well-Suited to Parallelize Optimization, in *Computational Intelligence in Expensive Optimization Problems; Adaption Learning and Optimization*. (eds. Tenne, Y., Goh, C.-K.) 131–162, 10.1007/978-3-642-10701-6_6 (Springer, Berlin, Heidelberg; 2010).

[57] Cox, D. D. & John, S. A Statistical Method for Global Optimization. *[Proceedings] 1992 IEEE International Conference on Systems, Man, and Cybernetics.* **2**, 1241–1246, 10.1109/ICSMC.1992.271617 (1992).

[58] Gutmann, M. U. & Corander, J. Bayesian Optimization for Likelihood-Free Inference of Simulator-Based Statistical Models. *Journal of Machine Learning Research.* **17**, 1–47 (2016).

[59] Alopaeus, M. *et al*. SP-LCC — a Dataset on the Structure and Properties of Lignin-Carbohydrate Complexes from Hardwood. *figshare*10.6084/m9.figshare.28444583 (2025). 10.1038/s41597-025-05327-8 PMC12166049 40514352

[60] Balakshin, M. Y. & Capanema, E. A. Comprehensive Structural Analysis of Biorefinery Lignins with a Quantitative 13C NMR Approach. *RSC Adv.* **5**, 87187–87199, 10.1039/C5RA16649G (2015).

[61] Gidh, A. V., Decker, S. R., Vinzant, T. B., Himmel, M. E. & Williford, C. Determination of Lignin by Size Exclusion Chromatography Using Multi Angle Laser Light Scattering. *J. Chromatogr. A.* **1114**, 102–110, 10.1016/j.chroma.2006.02.044 (2006). 16566937 10.1016/j.chroma.2006.02.044

[62] Matson, J. B., Steele, A. Q., Mase, J. D., Schulz, M. D. Polymer Characterization by Size-Exclusion Chromatography with Multi-Angle Light Scattering (SEC-MALS): A Tutorial Review. *Polym. Chem*. 2024, **15**, 127–142, 10.1039/D3PY01181J (2024). 10.1039/d3py01181j PMC11281244 39070757

