# Supplementary material for: SP-LCC — a dataset on the structure and properties of lignin-carbohydrate complexes from hardwood
Source: Sci Data. 2025 Jun 13;12:996. doi: 10.1038/s41597-025-05327-8 (PMC12166049; doi:10.1038/s41597-025-05327-8)
Supplement: Supplementary file 1 — SP-LCC — Supplementary Information [file 41597_2025_5327_MOESM1_ESM.docx]

**Electronic Supplementary Information**

**SP-LCC — a dataset on the structure and properties of lignin-carbohydrate complexes from hardwood**

Marie Alopaeus^1^, Matthias Stosiek^2,3,4^, Daryna Diment^5^, Joakim Löfgren^4^, MiJung Cho^5^, Jarl Hemming^1^, Teija Tirri^1^, Andrey Pranovich^1^, Patrik C. Eklund^6^, Davide Rigo^5^, Mikhail Balakshin^†,5^, Chunlin Xu^*1^ & Patrick Rinke^*2,3,4^

^1^ Laboratory of Natural Materials Technology, Åbo Akademi University, Henrikinkatu 2, 20500 Turku, Finland

^2^ Department of Physics, Technical University Munich, James-Franck-Str. 1, 85748 Garching, Germany

^3^ Atomistic Modelling Center, Munich Data Science Institute, Technical University Munich, Walther-von-Dyck-Straße 10, 85748 Garching, Germany

^4^ Department of Applied Physics, School of Science, Otakaari 1, Aalto University, 02150 Espoo, Finland

^5^ Department of Bioproducts and Biosystems, School of Chemical Engineering, Aalto University, Vuorimiehentie 1, 02150 Espoo, Finland

^6^ Laboratory of Molecular Science and Engineering, Åbo Akademi University, Henrikinkatu 2, 20500 Turku, Finland

† Deceased, 2022

*Corresponding authors: [patrick.rinke@tum.de](mailto:patrick.rinke@tum.de)
 [chunlin.xu@abo.fi](mailto:chunlin.xu@abo.fi)

**Table of contents**

1. **Experimental procedure on normalized radical scavenging index method.**
2. **Additional information on chromatogram and thermogram data.**
3. **Calculations for technical validation related to 2D HSQC NMR quantification of lignin moieties.**
4. **Standard deviation of selected moieties characterized by 2D HSQC NMR.**
5. **Outlier data points in surface tension measurements.**
6. **Values for the calculations of the average standard deviation of the physiochemical properties for technical validation.**
7. **Comparison of the determined physiochemical properties in SP-LCC to literature.**
8. **Chromatograms and thermograms for technical validation of molar mass analysis, thermal degradation and glass transition temperature.**
9. **References**

**Tables**

**Table S1.** Evaluation of the experimental errors related to the NMR quantification of lignin moieties. The experiments were repeated on four samples synthesized under the same conditions (P-factor = 500, L:S = 1, T = 195 °C, Sawdust (dry matter) = 4g). The moieties are expressed per 100 Ar units.

**Table S2.** Evaluation of measurement uncertainty related to physicochemical properties, four parallel measurements of each property measurement were conducted, and the average standard deviation was calculated.

**Table S3.** Evaluation of measurement uncertainty related to physicochemical properties, four parallel measurements of each property measurement were conducted, and the average relative standard deviation was calculated.

**Table S4.** A comparison of detected physiochemical property values in our AEL samples included in the SP-LCC compared to values reported for technical lignins in literature.

**Figures**

**Fig. S1.** Measurement uncertainties of for four selected lignin moieties, where y-axis describes the moiety content per 100 Ar and the x-axis describe the type of moiety. The error bars indicate the mean 1 standard deviation and were calculated from 4 repeated measurements (crosses) at each concentration level.

**Fig. S2.** Surface tension measurements showing the outliers in some of the measured concentrations (a) compared to the curves obtained from the average of four parallel measurements of the same samples used for the technical validation (b).

**Fig. S3.** Technical validation of refractive index chromatograms obtained from size exclusion chromatography equipped with refractive index detector. The shaded area in the chromatograms describes the SD.

**Fig. S4.** Technical validation of light scattering chromatograms obtained from size exclusion chromatography equipped with multi-angle light scattering detector. The shaded area in the chromatograms describes the SD.

**Fig. S5.** Technical validation of thermal degradation thermograms obtained from thermal gravimetrical analysis measurement of four parallel measurements of four samples. The shaded area in the thermograms describes the SD.

**Fig. S6.** Technical validation of the thermograms obtained from MDSC measurements of four parallel measurements of four samples. The shaded area in the thermograms describes the SD.

1. **Experimental procedure on normalized radical scavenging index method.**

Normalized radical scavenging index (nRSI) method expresses the ability of lignin and lignin-carbohydrate complexes (LCCs) to scavenge 2,2-diphenyl-1-picrylhydrazyl (DPPH) radical. The amount of the scavenged DPPH radicals is depicted as a decrease in DPPH absorbance from 0 time point to 24 h time point and is reflected in the form of inhibition percentage (IP) (Eq. S1). To eliminate possible experimental error, we separately measured DPPH degradation in 90 vol% acetone (aq.) as the difference between the absorbances at 0 time point and 24 h time point without the addition of lignin to the experimental tube (blank solution). We used the obtained difference as a correction factor added to the DPPH absorbance value in lignin-containing solutions at 24 h (Eq. S1).

$$\mathrm{IP}\left( \% \right)=\frac{{A_{0}(absorbance}_{t=0})-(A_{t}\left( \mathrm{absorbance}_{t} \right)+\left( A_{0DPPH}-A_{\mathrm{tDPPH}} \right))}{{A_{0}(absorbance}_{t=0})}\times100 (Eq.S1)$$

where $A_{0}$ is the absorbance of DPPH in lignin containing solution at the initial time point (t=0); $A_{t}$is the aborbance of DPPH in lignin-containing solution at the end-time point (24 h); $A_{0DPPH}$ is the DPPH absorbance in the blank solution (without lignin) at 0 time point; $A_{\mathrm{tDPPH}}$ is the DPPH absorbance in the blank solution at 24 h time point.

At the next stage, we plotted the IP(%) values as a function of the real lignin concentration (3-15 mg L^-1^). The data points were fitted using 2^nd^ order polynomial equation. The concentration of lignin corresponding to the IP value of 50% is the effective concentration (EC_50_), which was found as a solution of the 2^nd^ order polynomial equation. To ensure an accurate determination of antioxidant properties, EC_50_ value (mg L^-1^) was normalized by DPPH concentration (mmol L^-1^) (Eq. S2). The inversed value of the nEC_50_ represents the nRSI (mmol g^-1^) which indicates the amount of DPPH radicals in mmol that are scavenged by 1 g of lignin in 24 h (Eq. S3).

$$n\mathrm{EC}_{50}=\frac{\mathrm{EC}_{50}}{\left[ \mathrm{DPPH} \right]} (Eq.S2)$$

$$nRSI=\frac{1}{\mathrm{EC}_{50}} (Eq.S3)$$

1. **Additional information on chromatogram and thermogram data.**

For both the refractive index (RI) and light scattering (LS), the time of the measurements starts before 0 min. For example, for RI_Chromatogram_P813-169-1.56.csv and LS_Chromatogram_P813-169-1.56.csv the time begins at -0.26689 min and -0.06837 min respectively. This is due to the RI and LS detectors beginning their detections of analytes at different times. In addition to RI and LS detectors, the size exclusion chromatograph (SEC) is equipped with a UV detector. By default, this detector has been set to start at 0 min, and therefore, the starting time of RI and LS detection has been shifted so that all chromatograms are aligned. The UV detections are not representative and have, therefore, been excluded in this dataset.

For the thermograms obtained from the modulated differential scanning calorimetry (MDSC), the measurement begins after 0 min. For example, for Tg_Thermogram_P813-169-1.56 the measurement time begins at 29.19 min. This is due to the MDSC measurements consisting of three segments, and only the third segment is of interest as this contains the values for the glass transition temperature. Therefore, the two first segments have been excluded from this data set and the measurement time begins after 0 min. In addition, for the normalized reversing heat flow (column D) and normalized non-reversing heat flow (column E) the first several hundred rows are missing data points, whereas for normalized heat flow (column C) all rows contain data points. For example, for Tg_Thermogram_P813-169-1.56 the first data points for normalized reversing-heat flow and normalized non-reversing heat flow are given at row 595. This is due to the modulation method of the measurement. For the first two segments, a conventional DSC method is used, where only the normalized heat flow is measured. For the third segment, the method is switched to modulation mode, and in addition to the normalized heat flow, the normalized reversing heat flow and normalized non-reversing heat flow are measured. The measurements of the normalized heat flow are continuous, but for the other heat flows there is a delay in their measurement. The delay is necessary, as the Fourier deconvolution of the MDSC is only possible after a few periods of the sine wave modulation have passed. Therefore, there are no data points collected for the normalized reversing heat flow and the normalized non-reversing heat flow in the beginning of the segment.

For some of the thermograms obtained from the thermal gravimetric analysis (TGA), the values for the time of the measurement begin at values after 0 min. For example, for TD_Thermogram_P813-169-1.56.csv, the measurement time starts at 1.03 min. This is due to some measurements showing too much noise in the derivative weight curve affecting the determination of important parameters (onset temperature for thermal degradation and temperature at max degradation). Therefore, to reliably determine these parameters, the data points of the noise have been excluded from the data set and are shown as a jump in the measurement time. In addition, we note that the TGA instrument only outputs measurement times rounded to two decimal digits although multiple measurements per 0.01 minutes are executed. Due to this, the thermogram data contains multiple slightly varying weight measurement values for the same measurement time.

1. **Calculations for technical validation related to 2D HSQC NMR quantification of lignin moieties.**

We calculated the standard deviation (SD) and relative standard deviation (RSD) according to the following equations:

$$\bar{x}=\frac{\sum_{i=1}^{n_{1}} x_{i}}{n_{1}} \left( Eq. S4 \right)$$

$$s^{2}=\frac{\sum_{i=1}^{n_{1}} {(x_{i}-\bar{x})}^{2}}{n_{1}-1} (Eq. S5)$$

$$SD= \sqrt{s^{2}} (Eq. S6)$$

$$RSD = \frac{SD}{\bar{x}} \times100 (Eq. S7)$$

 where $\bar{x}$ is the mean of the parallel measurement for one sample, $x_{i}$ is the parallel measurements for one sample, $n_{1}$ is the number of parallel measurements for one sample, and $s^{2}$ is the variance of the parallel measurements for one sample.

**Table S1.** Evaluation of the experimental errors related to the NMR quantification of lignin moieties. The experiments were repeated on four samples synthesized under the same conditions (P-factor = 500, L:S = 1, T = 195 °C, Sawdust (dry matter) = 4g). The moieties are expressed per 100 Ar units.

| **Entry** | **Lignin moiety** | **Integration range** | **Experiment** | | | | **Mean** | **SD** | **RSD (%)** |
| --- | --- | --- | --- | --- | --- | --- | --- | --- | --- |
|  |  |  | **1** | **2** | **3** | **4** |  |  |  |
| 1 | S/G ratio |  | 2.41 | 2.42 | 2.41 | 2.45 | 2.42 | 0.02 | 0.78 |
| 2 | Acetyl groups | 22.2–18.9/2.15–1.68 | 6.20 | 5.70 | 6.20 | 6.00 | 6.03 | 0.24 | 3.92 |
| 3 | Alkyl-Alkyl | 45.8–42.5/3.09–2.88  51.1–46.5/2.83–2.50 | 1.65 | 1.64 | 1.65 | 1.77 | 1.68 | 0.06 | 3.69 |
| 4 | Alkyl-Aryl | 52.5–50.3/3.72–3.53  50.7–46.7/3.25–3.05  50.0–48.1/3.45–3.27  49.9–43.0/3.89–3.49 | 2.00 | 2.20 | 2.00 | 1.70 | 1.98 | 0.21 | 10.44 |
| 5 | γ-esters | 64.6–61.0/4.43–4.21 | 0.94 | 0.94 | 0.94 | 0.96 | 0.95 | 0.01 | 1.06 |
| 6 | β-O-4/α-OH | 73.7–69.7/5.26–4.65 | 20.60 | 20.30 | 20.60 | 21.70 | 20.80 | 0.62 | 2.96 |
| 7 | BE total | 79.0-82.0/4.5-5.1 | 3.30 | 3.30 | 3.30 | 3.10 | 3.25 | 0.10 | 3.08 |
| 8 | α-CO/β-O-4 | 84.5–82.0/5.36–5.06 | 1.10 | 1.13 | 1.10 | 1.08 | 1.10 | 0.02 | 1.87 |
| 9 | Resinol (β-β) | 86.7–84.1/4.71–4.52 | 5.84 | 5.84 | 5.84 | 5.90 | 5.86 | 0.03 | 0.51 |
| 10 | Phenylcoumaran (β-5) | 89.4–85.4/5.72–5.29 | 2.50 | 2.50 | 2.50 | 2.40 | 2.48 | 0.05 | 2.02 |
| 11 | GlcU Acid | 98.1–96.2/5.34–5.04 | 0.65 | 0.59 | 0.65 | 0.60 | 0.62 | 0.03 | 5.14 |
| 12 | GlcU Esters | 101.5–100.0/4.72–4.59 | 0.51 | 0.49 | 0.54 | 0.50 | 0.51 | 0.02 | 4.24 |
| 13 | PhGly | 104.7–99.5/5.23–4.80 | 0.34 | 0.35 | 0.34 | 0.29 | 0.33 | 0.03 | 8.21 |
| 14 | Term carb. | 94.5–90.0/5.1–4.8  98.6–95.4/5.3–5.0  99.1–94.7/4.5–4.15 | 2.20 | 2.00 | 2.20 | 2.00 | 2.10 | 0.12 | 5.50 |
| 15 | Internal carb. | 98.6–95.8/4.9–4.5  105.4–98.9/5.3–3.8  107.7–103.7/5.4–5.2 | 11.80 | 10.60 | 11.90 | 11.70 | 11.50 | 0.61 | 5.27 |
| 16 | Total carb.^a^ |  | 14.1 | 12.6 | 14.10 | 13.60 | 13.60 | 0.71 | 5.20 |
| 17 | Carb. DP^b^ |  | 6.40 | 6.30 | 6.40 | 6.80 | 6.48 | 0.22 | 3.42 |
| 18 | Conjugated S | 109.0–103.1/7.60–6.94 | 11.10 | 11.40 | 11.10 | 9.70 | 10.83 | 0.76 | 7.05 |
| 19 | Conj COOH | 124.1–121.0/7.6–7.4 | 1.10 | 1.10 | 1.10 | 1.00 | 1.08 | 0.05 | 4.65 |
| 20 | Conjugated G | 126.6–121.4/7.70–7.08 | 4.10 | 4.10 | 4.10 | 3.90 | 4.05 | 0.10 | 2.47 |
| 21 | Vinyl-Ar | 133.4–126.0/7.4–6.8 | 19.10 | 18.90 | 19.10 | 20.40 | 19.38 | 0.69 | 3.56 |
| 22 | Vinyl-Alk | 135.5–123.0/6.5–5.8 | 6.50 | 6.50 | 6.50 | 6.80 | 6.58 | 0.15 | 2.28 |
| 23 | Vinyl Ox | 149.5–137.2/8.25–7.20 | 1.98 | 1.97 | 1.98 | 1.95 | 1.97 | 0.01 | 0.72 |
| 24 | Fur-CHO | 179.6–176.1/9.68–9.40 | 0.19 | 0.19 | 0.19 | 0.15 | 0.18 | 0.02 | 11.11 |
| 25 | Ar-CHO | 192.5–189.2/10.06–9.55 | 1.60 | 1.60 | 1.50 | 1.10 | 1.45 | 0.24 | 16.42 |
| 26 | **Average RSD** | | | | | | | | **4.62** |

^a^Internal+terminal carbohydrates. ^b^ Average carbohydrates chain length calculated as the ratio between total and terminal carbohydrates.^10,30^ SD = standard deviation. RSD = relative standard deviation.

1. **Standard deviation of selected moieties characterized by 2D HSQC NMR.**


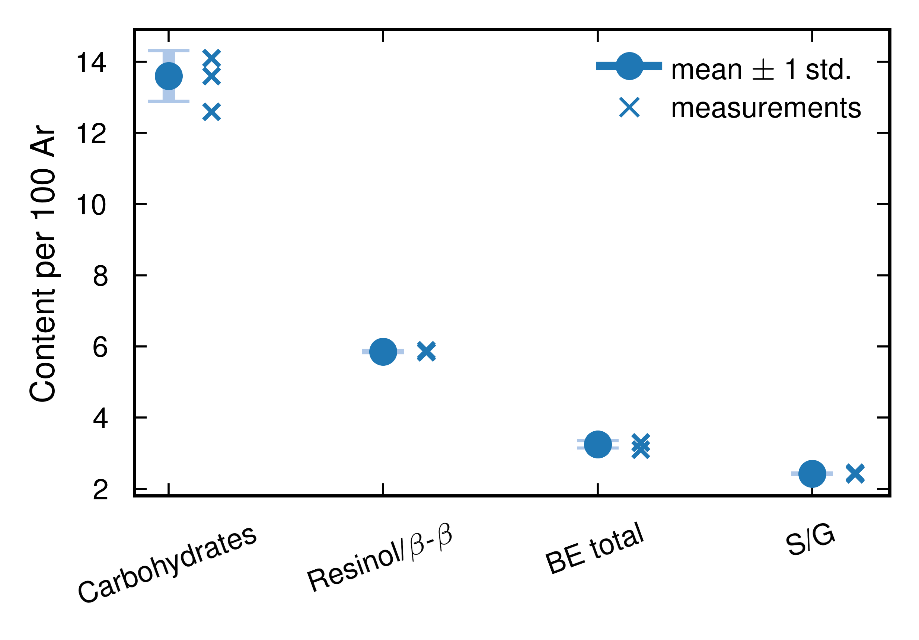


**Fig. S1.** Measurement uncertainties of for four selected lignin moieties, where y-axis describes the moiety content per 100 Ar and the x-axis describe the type of moiety. The error bars indicate the mean 1 standard deviation and were calculated from 4 repeated measurements (crosses) at each concentration level.

1. **Outlier data points in surface tension measurements.**

| **** | **** |
| --- | --- |

**Fig. S2.** Surface tension measurements showing (a) the outliers in some of the measured concentrations compared to (b) the curves obtained from the average of four parallel measurements of the same samples used for the technical validation.

1. **Values for calculating the average standard deviation of the physiochemical properties for technical validation.**

The average SD for each physicochemical property was calculated according to Eq. S5 described previously and Eq. S8 and S9 described below:

$$\bar{x}_{s^{2}}=\frac{\sum_{i=1}^{n_{2}} s^{2}}{n_{2}} (Eq. S8)$$

$$Avg. SD=\sqrt{\bar{x}_{s^{2}}} (Eq. S9)$$

where $\bar{x}_{s^{2}}$ is the mean of the variances for one property, $n_{2}$ is the number of variances per property. All values used for calculating the average SD is presented in Table S2.

**Table S2.** Evaluation of measurement uncertainty related to physicochemical properties, four parallel measurements of each property measurement were conducted, and the average standard deviation was calculated.

| **Property** | **Sample nr and ID** | **Measurement values** | **Variance** | **Average variance** | **Average SD** |
| --- | --- | --- | --- | --- | --- |
| **MWD [kDa]** |  |  |  |  |  |
| M_n_ | 17-I, P888-167-1.14 | 7.2, 8.0, 6.3, 6.2 | 0.72 |  |  |
|  | 50-I, P948-195-1.13 | 10.1, 9.2, 7.6, 7.4 | 1.68 |  |  |
|  | 58-I, P1000-195-0.73 | 7.2, 7.1, 6.2, 6.7 | 0.21 |  |  |
|  | 62-I, P250-185-1.26 | 4.8, 6.0, 4.7, 4.6 | 0.43 | 0.76 | 0.87 |
| M_p_ | 17-I, P888-167-1.14 | 5.5, 5.5, 6.3, 6.2 | 0.19 |  |  |
|  | 50-I, P948-195-1.13 | 6.0, 6.5, 6.7, 6.8 | 0.13 |  |  |
|  | 58-I, P1000-195-0.73 | 6.0, 5.6, 5.7, 6.8 | 0.30 |  |  |
|  | 62-I, P250-185-1.26 | 4.2, 4.6, 4.0, 4.5 | 0.08 | 0.17 | 0.41 |
| M_W_ | 17-I, P888-167-1.14 | 39.4, 37.0, 49.0, 49.5 | 41.70 |  |  |
|  | 50-I, P948-195-1.13 | 52.5, 45.4, 56.9, 53.3 | 23.17 |  |  |
|  | 58-I, P1000-195-0.73 | 24.4, 22.6, 35.5, 34.0 | 43.10 |  |  |
|  | 62-I, P250-185-1.26 | 7.4, 11.0, 9.9, 8.8 | 2.37 | 27.59 | 5.25 |
| **nRSI [mmol g^-1^]** |  |  |  |  |  |
|  | 2, P625-178-1.13 | 6.54, 6.86, 6.25, 7.09 | 0.13 |  |  |
|  | 3, P813-169-1.56 | 7.21, 7.30, 7.15, 8.13 | 0.21 |  |  |
|  | 5, P531-173-1.34 | 6.00, 6.42, 7.44, 7.54 | 0.58 |  |  |
|  | 6, P906-191-0.47 | 7.16, 7.12, 7.34, 8.35 | 0.34 | 0.31 | 0.56 |
| ***T*_g_ [°C]** |  |  |  |  |  |
|  | 49-I P1000-195-1.20 | 125.05, 124.22, 124.77, 125.69 | 0.38 |  |  |
|  | 58-I, P1000-195-0.73 | 122.84, 122.38, 123.30, 122.54 | 0.16 |  |  |
|  | 67-I, P993-195-1.24 | 129.22, 133.30, 132.53, 132.10 | 3.18 |  |  |
|  | 69-I, P840-195-2.00 | 122.25, 122.44, 122.35, 122.12 | 0.02 | 0.93 | 0.97 |
| **Thermal degradation [°C]** |  |  |  |  |  |
| *T*_onset_ | 42-I, P1000-195-1.61 | 189.19, 192.99, 193.41, 192.42 | 3.68 |  |  |
|  | 49-I, P1000-195-1.20 | 190.55, 190.80, 192.43, 191.96 | 0.82 |  |  |
|  | 67-I, P993-195-1.24 | 193.97, 192.43, 191.92, 199.34 | 11.54 |  |  |
|  | 69-I, P840-195-2.00 | 186.67, 188.14, 182.54, 189.64 | 9.00 | 6.26 | 2.50 |
| *T*_max_ | 42-I, P1000-195-1.61 | 363.39, 369.58, 369.75, 368.78 | 9.12 |  |  |
|  | 49-I, P1000-195-1.20 | 371.04, 372.67, 372.67, 374.34 | 1.82 |  |  |
|  | 67-I, P993-195-1.24 | 374.34, 375.81, 367.45, 373.58 | 13.55 |  |  |
|  | 69-I, P840-195-2.00 | 374.50, 371.30, 377.51, 380.80 | 16.55 | 10.26 | 3.20 |
| *T*_50_ | 42-I, P1000-195-1.61 | 423.22, 422.60, 429.84, 430.85 | 18.66 |  |  |
|  | 49-I, P1000-195-1.20 | 419.36, 418.35, 423.05, 427.65 | 17.76 |  |  |
|  | 67-I, P993-195-1.24 | 435.40, 451.68, 434.02, 452.95 | 103.90 |  |  |
|  | 69-I, P840-195-2.00 | 423.13, 422.38, 419.12, 416.92 | 8.37 | 37.17 | 6.10 |
| Char yield [%] | 42-I, P1000-195-1.61 | 35.98, 34.76, 36.80, 36.94 | 1.00 |  |  |
|  | 49-I, P1000-195-1.20 | 34.13, 34.96, 36.16, 37.12 | 1.56 |  |  |
|  | 67-I, P993-195-1.24 | 37.62, 40.07, 37.52, 40.13 | 2.14 |  |  |
|  | 69-I, P840-195-2.00 | 36.21, 36.03, 35.44, 34.97 | 0.32 | 1.25 | 1.12 |
| **Surface tension [mN m^-1^]** |  |  |  |  |  |
| 0.8 mg mL^-1^ | 6, P906-191-0.47 | 68.58, 68.49, 68.58, 64.37 | 4.38 |  |  |
|  | 41-I, P1000-195-0.96 | 68.37, 68.57, 69.94, 68.26 | 0.61 |  |  |
|  | 55-I, P849-178-0.77 | 69.39, 68.41, 69.03, 67.39 | 0.76 |  |  |
|  | 57-I, P878-195-0.82 | 68.16, 66.62, 66.52, 68.16 | 0.56 |  |  |
| 0.1 mg mL^-1^ | 6, P906-191-0.47 | 68.49, 68.74, 68.78, 67.22 | 0.54 |  |  |
|  | 41-I, P1000-195-0.96 | 68.51, 68.71, 68.57, 68.62 | 0.01 |  |  |
|  | 55-I, P849-178-0.77 | 69.06, 70.11, 68.82, 66.53 | 2.27 |  |  |
|  | 57-I, P878-195-0.82 | 68.15, 68.19, 68.74, 67.60 | 0.21 |  |  |
| 0.25 mg mL^-1^ | 6, P906-191-0.47 | 64.70, 64.44, 63.69, 64.02 | 0.20 |  |  |
|  | 41-I, P1000-195-0.96 | 64.82, 63.72, 63.91, 65.37 | 0.60 |  |  |
|  | 55-I, P849-178-0.77 | 65.49, 65.93, 64.22, 63.85 | 0.99 |  |  |
|  | 57-I, P878-195-0.82 | 64.22, 63.69, 65.58, 63.25 | 0.60 |  |  |
| 0.4 mg mL^-1^ | 6, P906-191-0.47 | 60.50, 63.23, 61.33, 61.11 | 1.39 |  |  |
|  | 41-I, P1000-195-0.96 | 61.00, 62.48, 62.67, 62.34 | 0.58 |  |  |
|  | 55-I, P849-178-0.77 | 64.96, 63.23, 63.68, 61.80 | 1.70 |  |  |
|  | 57-I, P878-195-0.82 | 62.70, 61.93, 61.72, 61.90 | 0.19 |  |  |
| 0.5 mg mL^-1^ | 6, P906-191-0.47 | 61.53, 61.72, 61.61, 61.20 | 0.05 |  |  |
|  | 41-I, P1000-195-0.96 | 60.26, 61.22, 61.17, 61.47 | 0.28 |  |  |
|  | 55-I, P849-178-0.77 | 63.54, 64.56, 62.85, 62.67 | 0.73 |  |  |
|  | 57-I, P878-195-0.82 | 62.52, 62.47, 63.30, 62.68 | 0.12 | 0.86 | 0.93 |

The SD and RSD for each physicochemical property was calculated according to Eq. S4-S7:

**Table S3.** Evaluation of measurement uncertainty related to physicochemical properties, four parallel measurements of each property measurement were conducted, and the average relative standard deviation was calculated.

| **Property** | **Sample nr and ID** | **Measurement values** | **Mean** | **SD** | **RSD** | **Avg. RSD** |
| --- | --- | --- | --- | --- | --- | --- |
| **MWD [kDa]** |  |  |  |  |  |  |
| M_n_ | 17-I, P888-167-1.14 | 7.2, 8.0, 6.3, 6.2 | 6.93 | 0.85 | 12.22 |  |
|  | 50-I, P948-195-1.13 | 10.1, 9.2, 7.6, 7.4 | 8.58 | 1.30 | 15.13 |  |
|  | 58-I, P1000-195-0.73 | 7.2, 7.1, 6.2, 6.7 | 6.80 | 0.45 | 6.69 |  |
|  | 62-I, P250-185-1.26 | 4.8, 6.0, 4.7, 4.6 | 5.03 | 0.66 | 13.04 | 11.77 |
| M_p_ | 17-I, P888-167-1.14 | 5.5, 5.5, 6.3, 6.2 | 5.88 | 0.43 | 7.40 |  |
|  | 50-I, P948-195-1.13 | 6.0, 6.5, 6.7, 6.8 | 6.50 | 0.36 | 5.48 |  |
|  | 58-I, P1000-195-0.73 | 6.0, 5.6, 5.7, 6.8 | 6.03 | 0.54 | 9.03 |  |
|  | 62-I, P250-185-1.26 | 4.2, 4.6, 4.0, 4.5 | 4.33 | 0.28 | 6.37 | 7.07 |
| M_W_ | 17-I, P888-167-1.14 | 39.4, 37.0, 49.0, 49.5 | 43.73 | 6.46 | 14.77 |  |
|  | 50-I, P948-195-1.13 | 52.5, 45.4, 56.9, 53.3 | 52.03 | 4.81 | 9.25 |  |
|  | 58-I, P1000-195-0.73 | 24.4, 22.6, 35.5, 34.0 | 29.13 | 6.57 | 22.54 |  |
|  | 62-I, P250-185-1.26 | 7.4, 11.0, 9.9, 8.8 | 9.28 | 1.54 | 16.60 | 15.66 |
| **nRSI [mmol g^-1^]** |  |  |  |  |  |  |
|  | 2, P625-178-1.13 | 6.54, 6.86, 6.25, 7.09 | 6.69 | 0.37 | 5.48 |  |
|  | 3, P813-169-1.56 | 7.21, 7.30, 7.15, 8.13 | 7.45 | 0.46 | 6.17 |  |
|  | 5, P531-173-1.34 | 6.00, 6.42, 7.44, 7.54 | 6.85 | 0.76 | 11.09 |  |
|  | 6, P906-191-0.47 | 7.16, 7.12, 7.34, 8.35 | 7.49 | 0.58 | 7.74 | 7.62 |
| ***T*_g_ [°C]** |  |  |  |  |  |  |
|  | 49-I P1000-195-1.20 | 125.05, 124.22, 124.77, 125.69 | 124.93 | 0.61 | 0.49 |  |
|  | 58-I, P1000-195-0.73 | 122.84, 122.38, 123.30, 122.54 | 122.77 | 0.40 | 0.33 |  |
|  | 67-I, P993-195-1.24 | 129.22, 133.30, 132.53, 132.10 | 131.79 | 1.78 | 1.35 |  |
|  | 69-I, P840-195-2.00 | 122.25, 122.44, 122.35, 122.12 | 122.29 | 0.14 | 0.11 | 0.57 |
| **Thermal degradation [°C]** |  |  |  |  |  |  |
| *T*_onset_ | 42-I, P1000-195-1.61 | 189.19, 192.99, 193.41, 192.42 | 192.00 | 1.92 | 1.00 |  |
|  | 49-I, P1000-195-1.20 | 190.55, 190.80, 192.43, 191.96 | 191.44 | 0.90 | 0.47 |  |
|  | 67-I, P993-195-1.24 | 193.97, 192.43, 191.92, 199.34 | 194.42 | 3.40 | 1.75 |  |
|  | 69-I, P840-195-2.00 | 186.67, 188.14, 182.54, 189.64 | 186.70 | 3.00 | 1.61 | 1.21 |
| *T*_max_ | 42-I, P1000-195-1.61 | 363.39, 369.58, 369.75, 368.78 | 367.88 | 3.02 | 0.82 |  |
|  | 49-I, P1000-195-1.20 | 371.04, 372.67, 372.67, 374.34 | 372.68 | 1.35 | 0.36 |  |
|  | 67-I, P993-195-1.24 | 374.34, 375.81, 367.45, 373.58 | 372.80 | 3.68 | 0.99 |  |
|  | 69-I, P840-195-2.00 | 374.50, 371.30, 377.51, 380.80 | 376.03 | 4.07 | 1.08 | 0.81 |
| *T*_50_ | 42-I, P1000-195-1.61 | 423.22, 422.60, 429.84, 430.85 | 426.63 | 4.32 | 1.01 |  |
|  | 49-I, P1000-195-1.20 | 419.36, 418.35, 423.05, 427.65 | 422.10 | 4.21 | 1.00 |  |
|  | 67-I, P993-195-1.24 | 435.40, 451.68, 434.02, 452.95 | 443.51 | 10.19 | 2.30 |  |
|  | 69-I, P840-195-2.00 | 423.13, 422.38, 419.12, 416.92 | 420.39 | 2.89 | 0.69 | 1.25 |
| Char yield [%] | 42-I, P1000-195-1.61 | 35.98, 34.76, 36.80, 36.94 | 36.12 | 1.00 | 2.77 |  |
|  | 49-I, P1000-195-1.20 | 34.13, 34.96, 36.16, 37.12 | 35.64 | 1.25 | 3.50 |  |
|  | 67-I, P993-195-1.24 | 37.62, 40.07, 37.52, 40.13 | 38.83 | 1.46 | 3.77 |  |
|  | 69-I, P840-195-2.00 | 36.21, 36.03, 35.44, 34.97 | 35.66 | 0.57 | 1.59 | 2.91 |
| **Surface tension [mN m^-1^]** |  |  |  |  |  |  |
| 0.8 mg mL^-1^ | 6, P906-191-0.47 | 68.58, 68.49, 68.58, 64.37 | 67.50 | 2.09 | 3.10 |  |
|  | 41-I, P1000-195-0.96 | 68.37, 68.57, 69.94, 68.26 | 68.78 | 0.78 | 1.13 |  |
|  | 55-I, P849-178-0.77 | 69.39, 68.41, 69.03, 67.39 | 68.56 | 0.87 | 1.27 |  |
|  | 57-I, P878-195-0.82 | 68.16, 66.62, 66.52, 68.16 | 67.12 | 0.75 | 1.12 |  |
| 0.1 mg mL^-1^ | 6, P906-191-0.47 | 68.49, 68.74, 68.78, 67.22 | 68.31 | 0.74 | 1.08 |  |
|  | 41-I, P1000-195-0.96 | 68.51, 68.71, 68.57, 68.62 | 68.60 | 0.09 | 0.13 |  |
|  | 55-I, P849-178-0.77 | 69.06, 70.11, 68.82, 66.53 | 68.63 | 1.51 | 2.20 |  |
|  | 57-I, P878-195-0.82 | 68.15, 68.19, 68.74, 67.60 | 68.17 | 0.46 | 0.68 |  |
| 0.25 mg mL^-1^ | 6, P906-191-0.47 | 64.70, 64.44, 63.69, 64.02 | 64.21 | 0.45 | 0.70 |  |
|  | 41-I, P1000-195-0.96 | 64.82, 63.72, 63.91, 65.37 | 64.46 | 0.77 | 1.20 |  |
|  | 55-I, P849-178-0.77 | 65.49, 65.93, 64.22, 63.85 | 64.87 | 1.00 | 1.54 |  |
|  | 57-I, P878-195-0.82 | 64.22, 63.69, 65.58, 63.25 | 64.19 | 0.77 | 1.20 |  |
| 0.4 mg mL^-1^ | 6, P906-191-0.47 | 60.50, 63.23, 61.33, 61.11 | 61.54 | 1.18 | 1.91 |  |
|  | 41-I, P1000-195-0.96 | 61.00, 62.48, 62.67, 62.34 | 62.12 | 0.76 | 1.22 |  |
|  | 55-I, P849-178-0.77 | 64.96, 63.23, 63.68, 61.80 | 63.41 | 1.30 | 2.05 |  |
|  | 57-I, P878-195-0.82 | 62.70, 61.93, 61.72, 61.90 | 62.06 | 0.43 | 0.70 |  |
| 0.5 mg mL^-1^ | 6, P906-191-0.47 | 61.53, 61.72, 61.61, 61.20 | 61.52 | 0.22 | 0.36 |  |
|  | 41-I, P1000-195-0.96 | 60.26, 61.22, 61.17, 61.47 | 61.03 | 0.53 | 0.87 |  |
|  | 55-I, P849-178-0.77 | 63.54, 64.56, 62.85, 62.67 | 63.40 | 0.86 | 1.35 |  |
|  | 57-I, P878-195-0.82 | 62.52, 62.47, 63.30, 62.68 | 62.81 | 0.34 | 0.54 | 1.24 |

1. **Comparison of the determined physiochemical properties in SP-LCC to literature.**

**Table S4.** A comparison of detected physiochemical property values in our AEL samples included in the SP-LCC compared to values reported for technical lignins in literature.

| **Property** | **Region in SP-LCC dataset** | **Region in literature [ref]** |
| --- | --- | --- |
| **MMD [kDa]** |  |  |
| M_n_ | 3.60-8.40 | Indulin AT: 6.0^1^ |
|  |  | Lignoboost: 3.4^1^ |
|  |  | Organosolv: 3.3^1^ |
|  |  | Milled wood lignin: 5.5^1^ |
| M_W_ | 4.60-117.00 | Indulin AT: 14^1^ |
|  |  | Lignoboost: 16^1^ |
|  |  | Organosolv: 10^1^ |
|  |  | Milled wood lignin: 27^1^ |
| **nRSI [mmol g^-1^]** | 3.64-8.59 | Indulin AT: 8.1^2^ |
|  |  | Alcell: 9.86^a^ |
| ***T*_g_ [°C]** | 87.83-133.82 | Indulin AT: 156^2^, 147^3^ |
|  |  | Alcell: 91^4^, 100^5^ |
| **Thermal degradation [°C]** |  |  |
| *T*_onset_ | 148.47-197.38 | Indulin AT: 231^3^ |
|  |  | Kraft lignin: 156.6^6^ |
| *T*_max_ | 344.14-374.50 | Indulin AT: 384^3^ |
|  |  | Kraft lignin: 417.5^6^ |
|  |  | Alcell: 374^5^ |
|  |  | LCC: 256-276^7^ |
| *T*_50_ | 361.40-447.34 | Kraft lignin: 497.8^6^ |
| Char yield [%] | 26.89-39.19 | LCC: 16-31.2 |
| **Surface tension [mN m^-1^]** | 0.5 mg mL^-1^: 51.83-64.19 | 1. Indulin AT: 65 (0.3 mg mL^-1^)^4, b^ |
|  |  | Kraft lignin: >65^8^ |
|  |  | Alcell: 60.3 (0.3 mg mL^-1^)^4, b^ |

^a^Additional antioxidant activity measurement was performed on the organosolv lignin Alcell to obtain a reference value for the validation of our data.

^b^According to the authors, the correct value of the surface tension reported in the cited article should be 0.3 mg mL^-1^ and not 0.1 mg mL^-1^ as stated in the article.

1. **Chromatograms and thermograms for technical validation of molar mass analysis, thermal degradation and glass transition temperature**


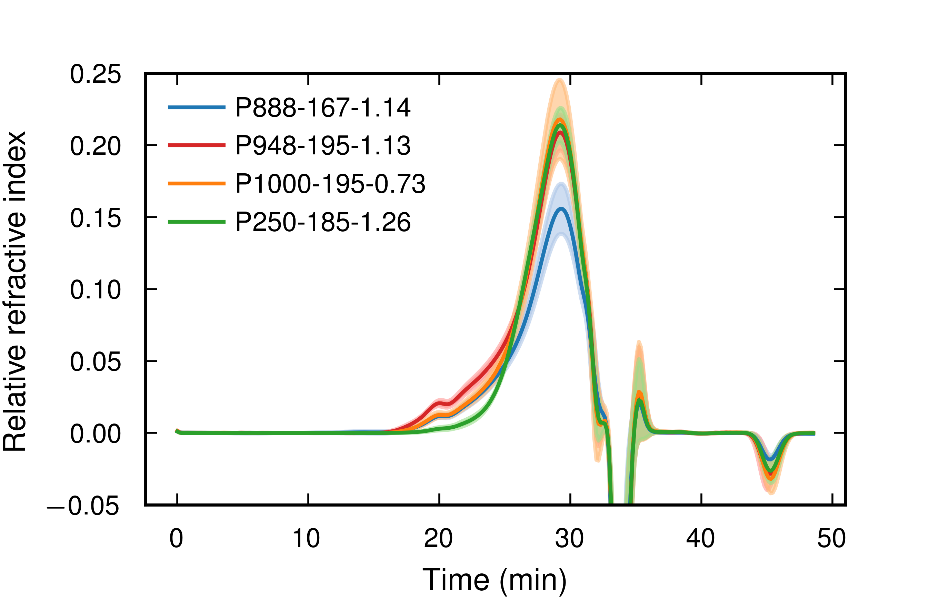


**Fig. S3.** Technical validation of refractive index chromatograms obtained from size exclusion chromatography equipped with refractive index detector. The shaded area in the chromatograms describes the SD.


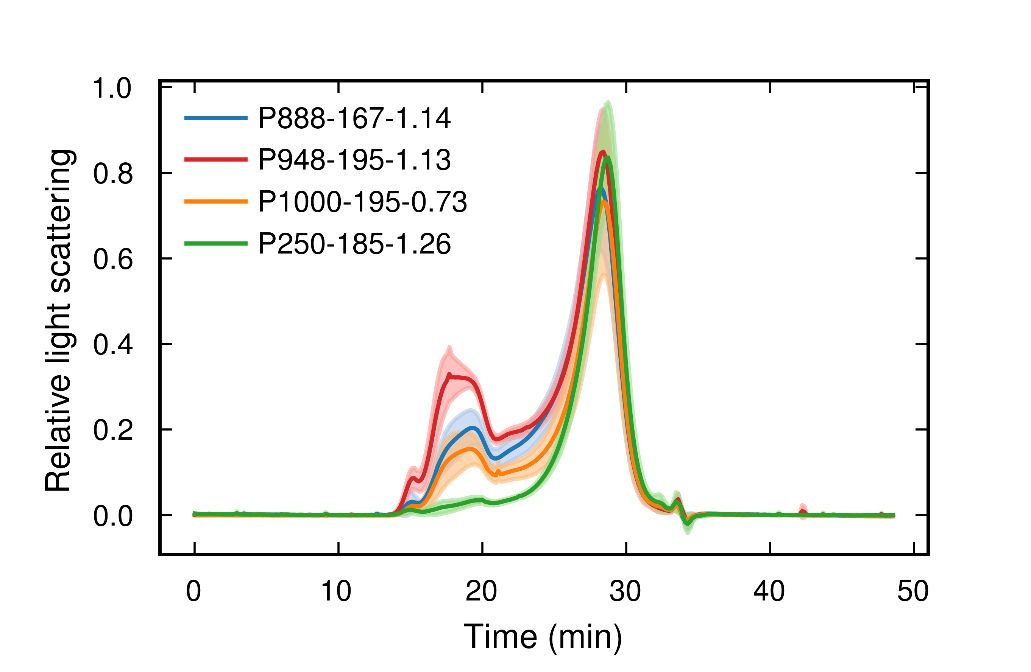


**Fig. S4.** Technical validation of light scattering chromatograms obtained from size exclusion chromatography equipped with multi-angle light scattering detector. The shaded area in the chromatograms describes the SD.

| 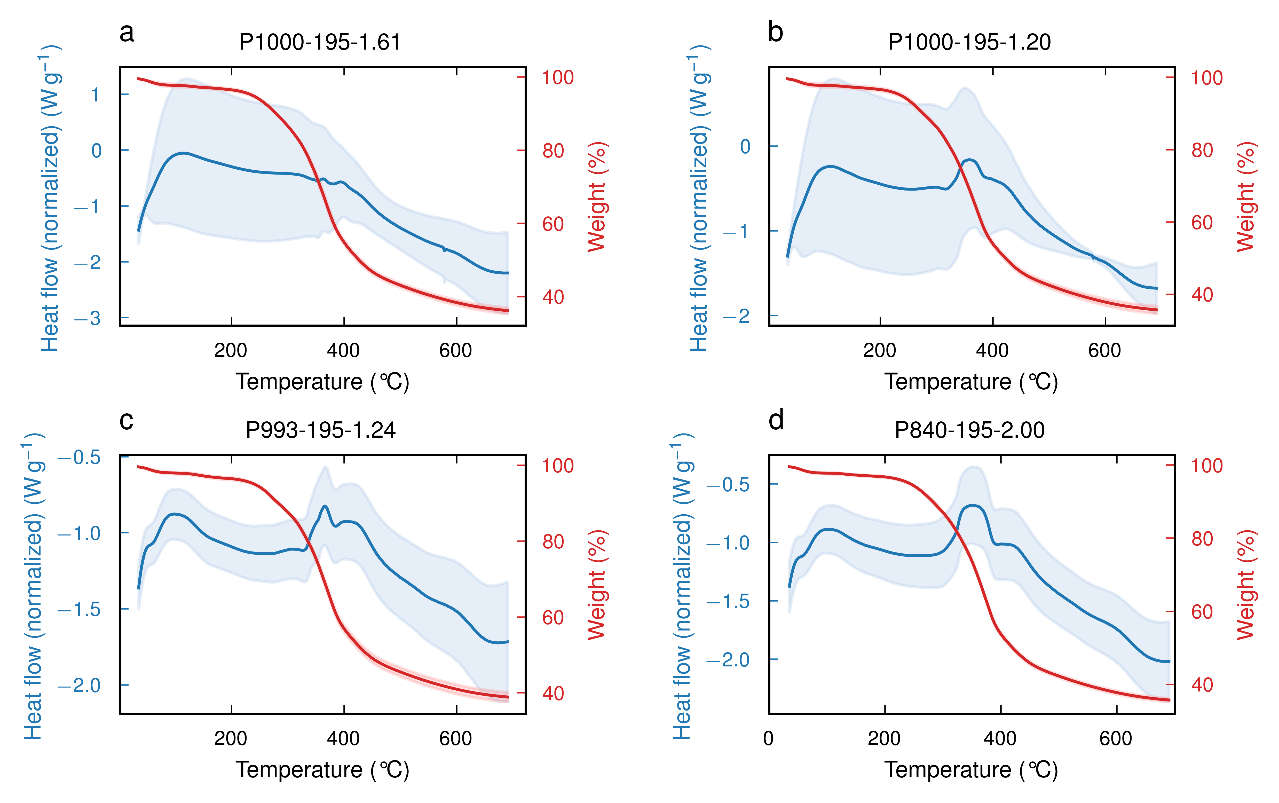 |
| --- |

**Fig. S5.** Technical validation of thermal degradation thermograms obtained from thermal gravimetrical analysis measurement of four parallel measurements of four samples. The shaded area in the thermograms describes the SD.

| 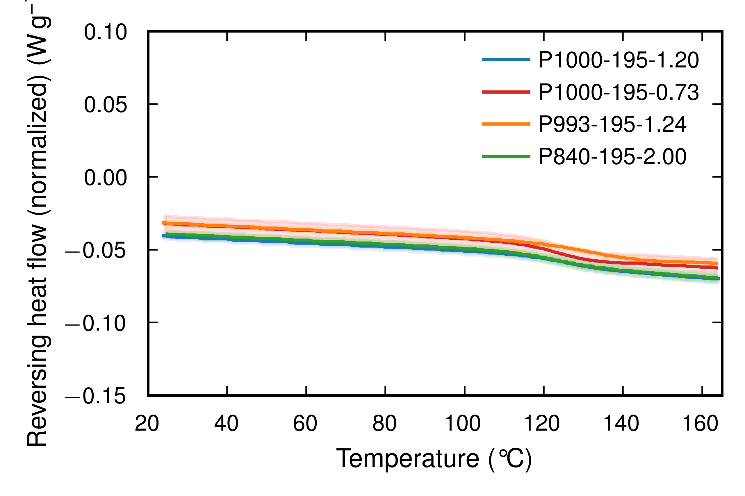 |
| --- |

**Fig. S6.** Technical validation of the thermograms obtained from MDSC measurements of four parallel measurements of four samples. The shaded area in the thermograms describes the SD.

1. **References**
2. Zinovyev, G.; Sulaeva, I.; Podzimek, S.; Rössner, D.; Kilpeläinen, I.; Sumerskii, I.; Rosenau, T.; Potthast, A. Getting Closer to Absolute Molar Masses of Technical Lignins. *ChemSusChem*. **11**, 3259–3268, <https://doi.org/10.1002/cssc.201801177> (2018).
3. Diment, D., Tkachenko, O., Schlee, P., Kohlhuber, N., Potthast, A., Budnyak, T. M., Rigo, D., Balakshin, M. Study toward a More Reliable Approach to Elucidate the Lignin Structure-Property-Performance Correlation. *Biomacromolecules*. **25**, 200–212, <https://doi.org/10.1021/acs.biomac.3c00906> (2024).
4. Li, H., McDonald, A. G. Fractionation and Characterization of Industrial Lignins. *Ind. Crops. Prod.* **62**, 67–76, <https://doi.org/10.1016/j.indcrop.2014.08.013> (2014).
5. Tarasov, D., Schlee, P., Pranovich, A., Moreno, A., Wang, L., Rigo, D., Sipponen, M. H., Xu, C., Balakshin, M. AqSO Biorefinery: A Green and Parameter-Controlled Process for the Production of Lignin–Carbohydrate Hybrid Materials. *Green Chem*. **24**, 6639–6656, <https://doi.org/10.1039/D2GC02171D> (2022).
6. Huijgen, W. J. J., Telysheva, G., Arshanitsa, A., Gosselink, R. J. A., de Wild, P. J. Characteristics of Wheat Straw Lignins from Ethanol-Based Organosolv Treatment. *Ind. Crops. Prod*. **59**, 85–95, <https://doi.org/10.1016/j.indcrop.2014.05.003> (2014).
7. Chemical modification of kraft lignin: Effect on chemical and thermal properties. *BioRes*. **9**, 5488-5500 (2014).
8. Su, C., Gan, T., Liu, Z., Chen, Y., Zhou, Q., Xia, J., Cao, Y. Enhancement of the Antioxidant Abilities of Lignin and Lignin-Carbohydrate Complex from Wheat Straw by Moderate Depolymerization via LiCl/DMSO Solvent Catalysis. *Int. J. Biol. Macromol*. **184**, 369–379, <https://doi.org/10.1016/j.ijbiomac.2021.06.063> (2021).
9. Li, S., Willoughby, J. A., Rojas, O. J. Oil-in-Water Emulsions Stabilized by Carboxymethylated Lignins: Properties and Energy Prospects. *ChemSusChem*. 2016, **9**, 2460–2469 (2016).
